# Supplementary material for: Local and global changes in cell density induce reorganisation of 3D packing in a proliferating epithelium
Source: Development. 2024 May 7;151(20):dev202362. doi: 10.1242/dev.202362 (PMC11112164; doi:10.1242/dev.202362)
Supplement: Supplementary information [file develop-151-202362-s1.pdf]

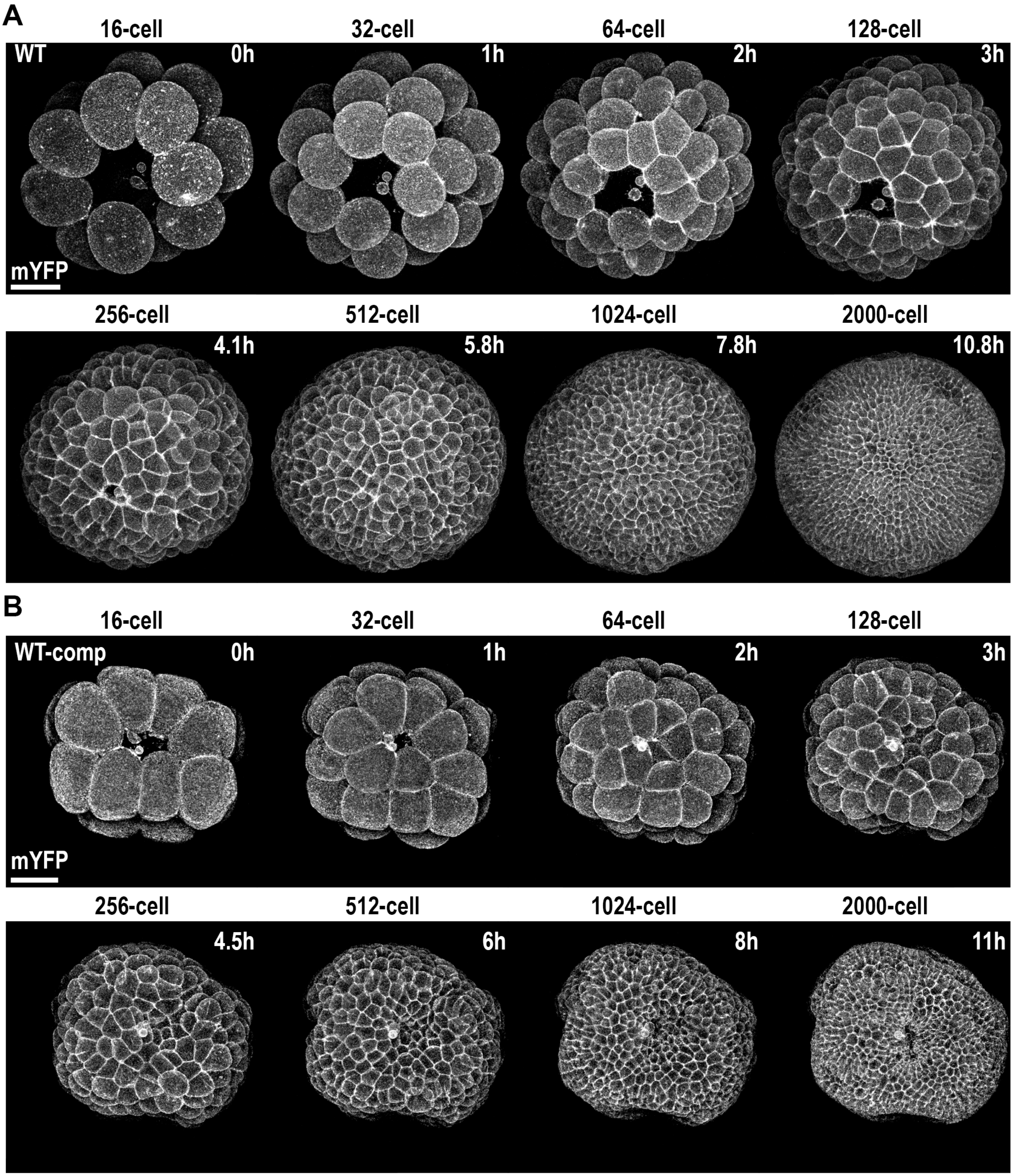

Fig. S1. *Patiria miniata* embryo development. Maximum projections of a representative WT sea star embryo (**A**) or WT-comp embryo (**B**) expressing the membrane marker mYFP at different stages from 16-cells to 2000-cells. Scale bars, 50 μm.

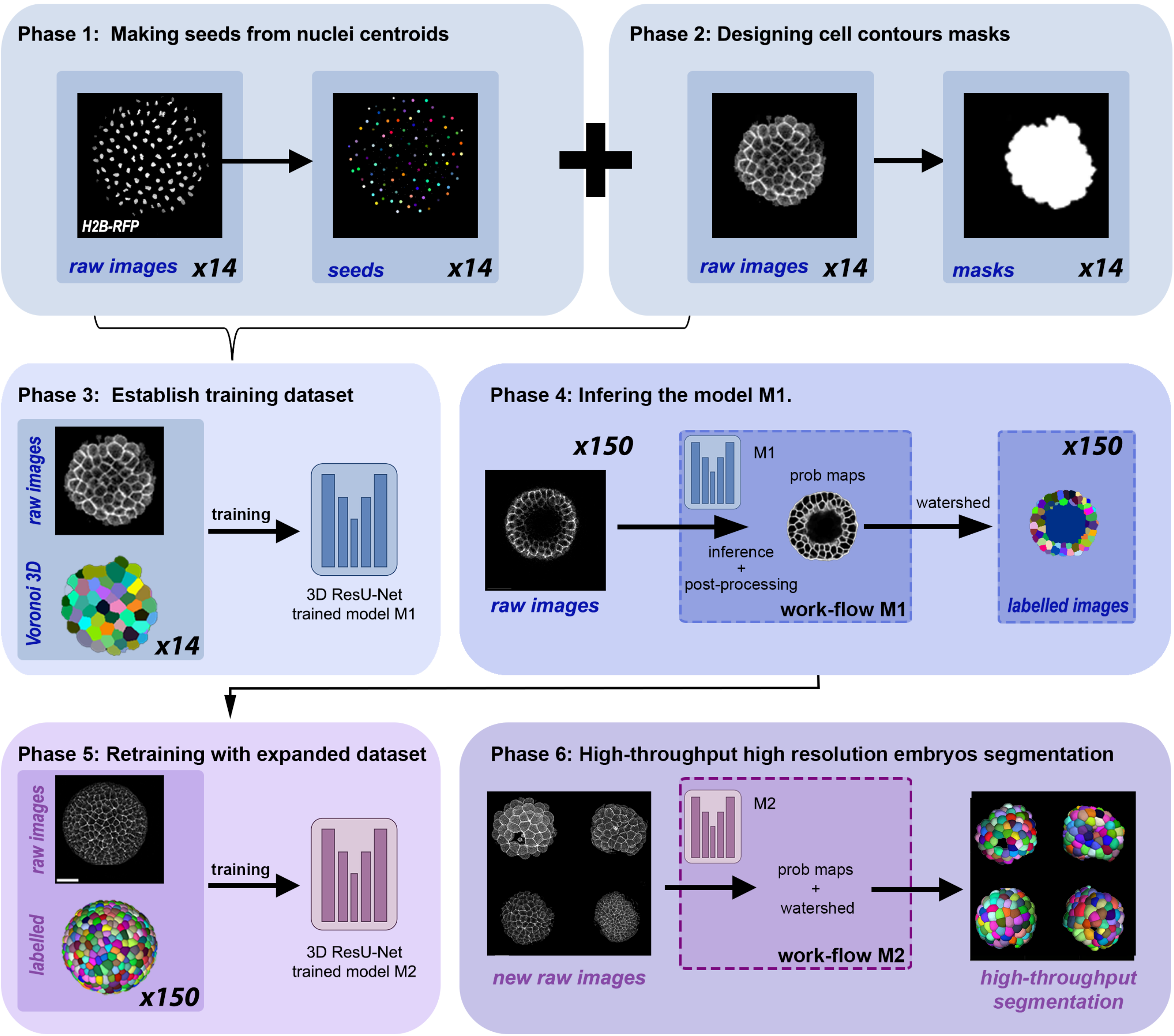

Fig. S2. Deep learning-based 3D segmentation. Workflow scheme showing the different steps followed to segment sea star embryos.

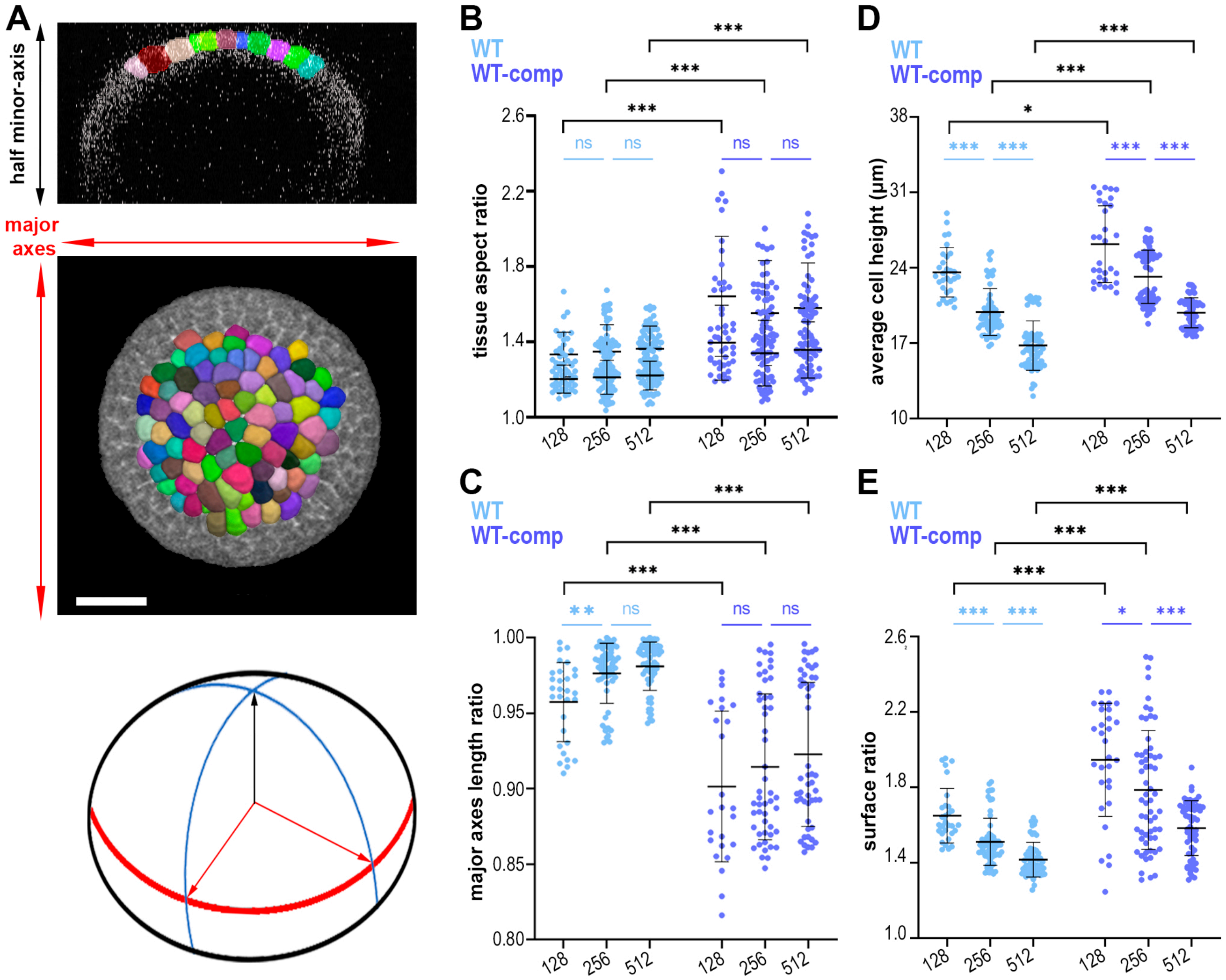

Fig. S3. Shape of WT and WT-comp embryos at 128-, 256- and 512-cells. A) Orthogonal view (top) and maximum projection (centre) of a representative WT half embryo. (Bottom) Estimate of the complete shape. Scale bars, 50 µm. Quantification of the tissue aspect ratio (**B**) and major axes length ratio (**C**) of the whole embryos. Quantification of the tissue average cell height (**D**) and surface ratio (**E**) of the segmented region. WT: n=150 time points, 6 embryos, 4 experiments. WT-comp: n=125 time points, 5 embryos, 4 experiments for B-C panels, n=150 time points, 6 embryos, 5 experiments for D-E panels. Mean ± s.d. Statistical tests: Mann-Whitney tests with Bonferroni multiple comparisons correction (black) and Kruskal-Wallis tests (blue) with Dunn multiple comparisons correction; ns: non-significant; *: p-value <0.05; **: p-value <0.01 ***: p-value <0.001.

Development • Supplementary information

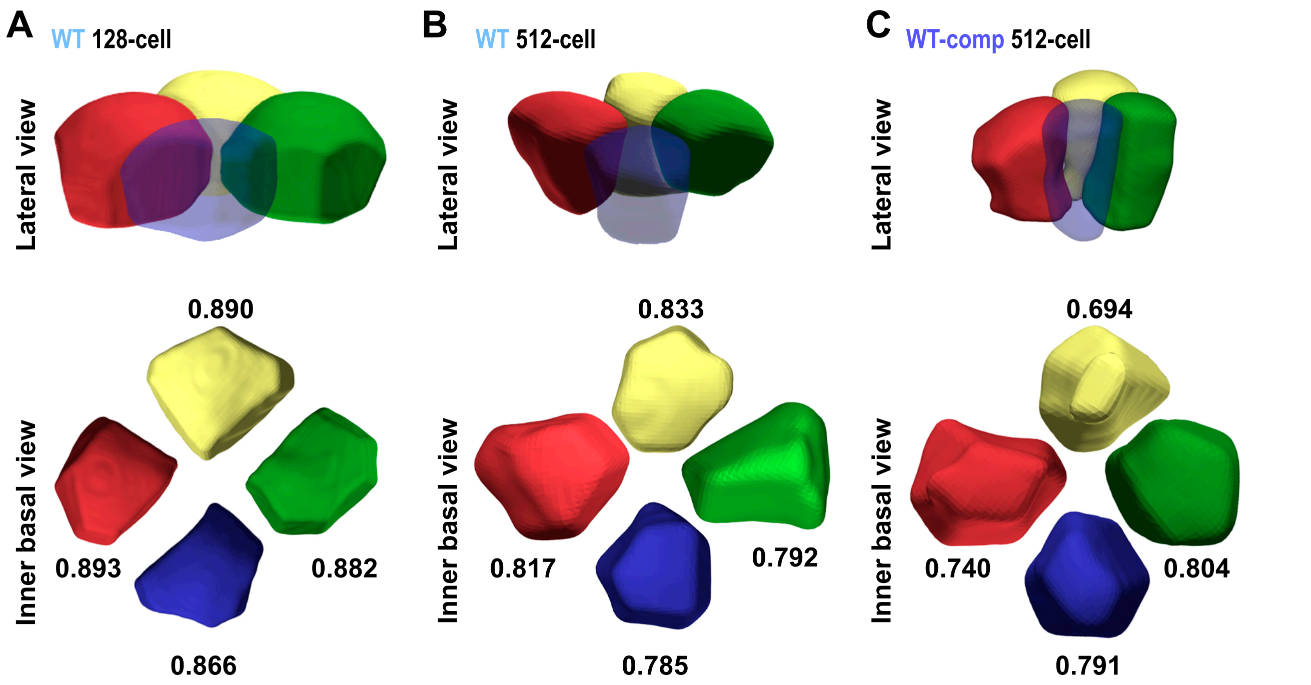

Fig. S4. Cell compaction. 3D representation of 4-cell motives from a lateral (Top) and inner (Bottom) view from 128-cell WT embryo (**A**) 512-cell WT embryo (**B**) 128-cell stage (**C**) WT-comp embryo. In the inner view, the numbers displayed next to each cell indicate the individual value of the convexity ratio (**see Materials and methods**).

Development • Supplementary information

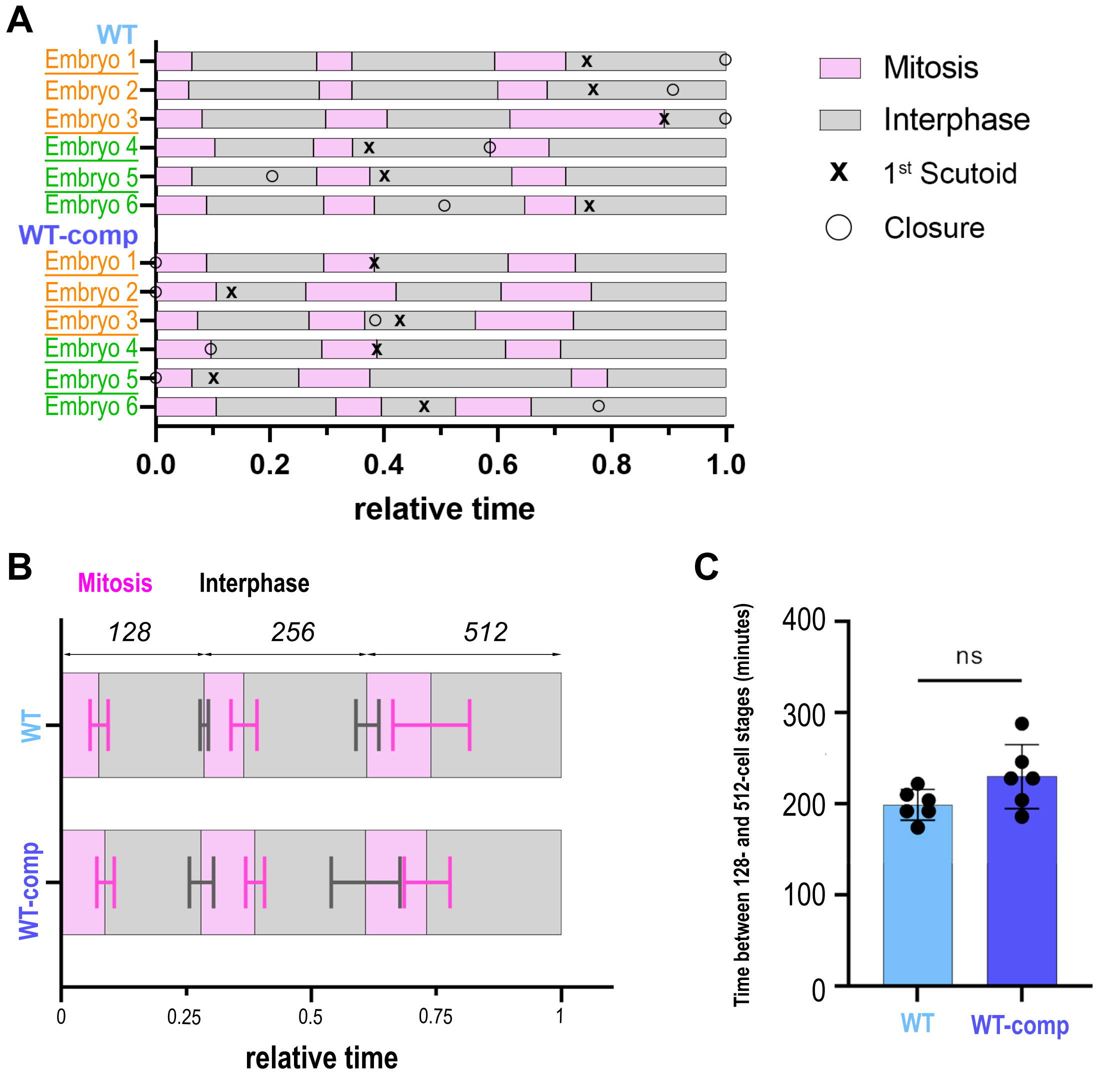

Fig. S5. Single-cell tracking of division: analysis of cell proliferation rate per embryo. **A**) Quantification of mitosis and interphase time intervals per embryo showing the moment the tissue is completely sealed and the first cell adopting scutoidal shape. Quantification of the average time elapsed among each interphase and mitosis intervals (**B**) and between the beginning of the 128-cell stage and the end of 512-cell stage (**C**) in WT and WT-comp embryos. WT: n=150 time points, 6 embryos, 4 experiments. WT-comp: n=150 time points, 6 embryos, 5 experiments. Mean ± s.d. Statistical tests: two-tailed Student t-test; ns: non-significant.

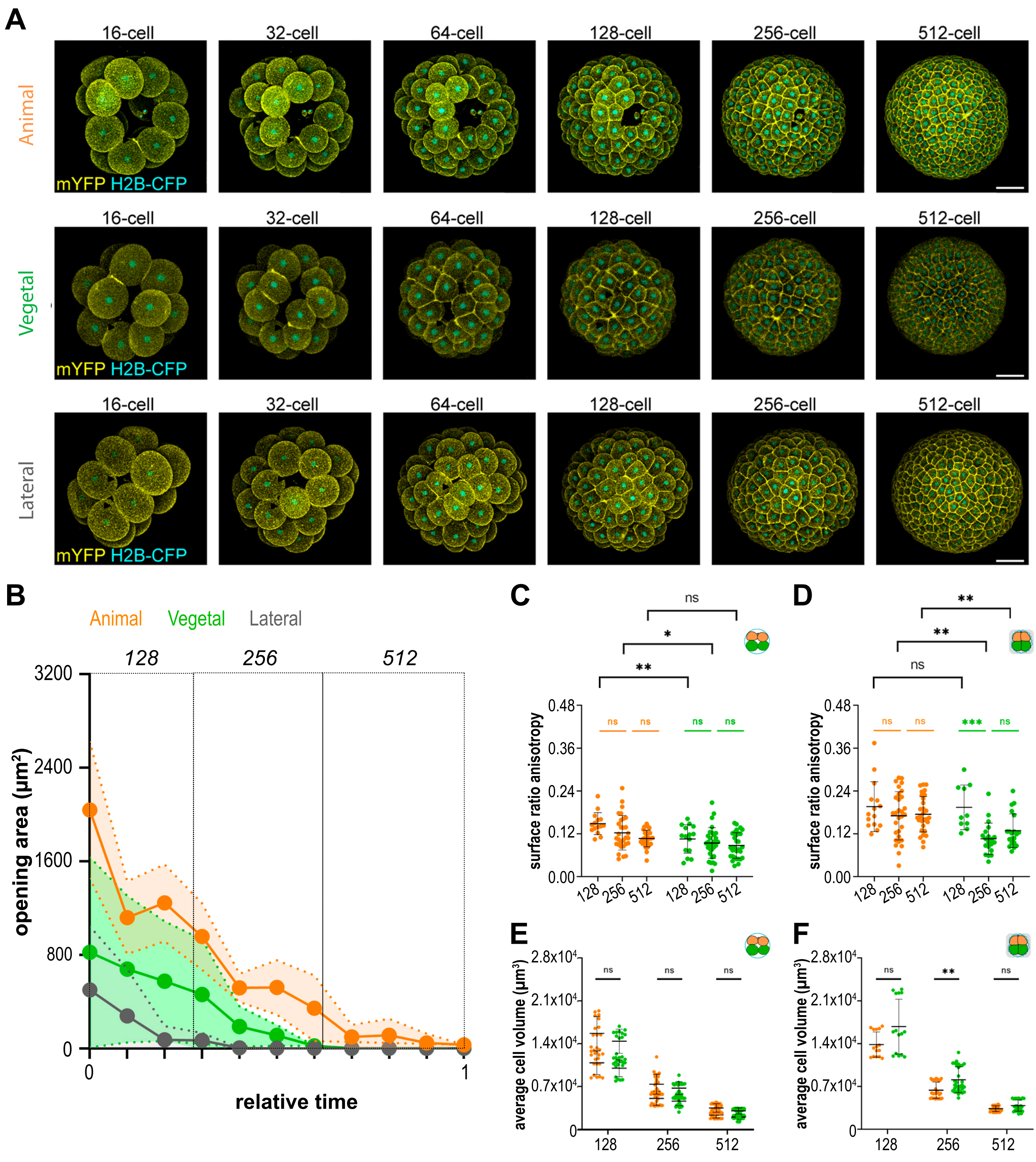

Fig. S6. Opening areas and compaction. A) Maximum projections of representative WT sea star embryos expressing a membrane marker (mYFP) and nuclear marker (H2B-CFP) from an animal (top), vegetal (centre) or lateral (bottom) view. Scale bars, 50 µm. **B**) Quantification of opening areas in animal, vegetal and lateral regions. WT animal: n= 6 embryos, WT vegetal: n= 5 embryos; WT lateral: n= 4 embryos; 5 experiments. Quantification of surface ratio anisotropy

Development • Supplementary information

in both regions of WT (**C**) and WT-comp (**D**). Quantification of cell volume in both regions of WT (**E**) and WT-comp (**F**). WT animal: n= 75 time points, 3 embryos, 3 experiments. WT vegetal: n= 75 time points, 3 embryos, 2 experiments. WT-comp animal: n= 75 time points, 3 embryos, 2 experiments. WT-comp vegetal: n= 50 time points, 2 embryos, 2 experiments for C-D panels, n= 75 time points, 3 embryos, 3 experiments for E-F panels. Mean ± s.d. Statistical tests Mann-Whitney tests with Bonferroni multiple comparisons correction (black) except in C where two-way ANOVA test with Bonferroni multiple comparisons correction was applied. Kruskal-Wallis tests with Dunn multiple comparisons correction (orange and green) except in C where one-way ANOVA tests with Tukey multiple comparisons correction was used; ns: non-significant; *: p-value <0.05; **: p-value <0.01; ***: p-value <0.001.

Development • Supplementary information

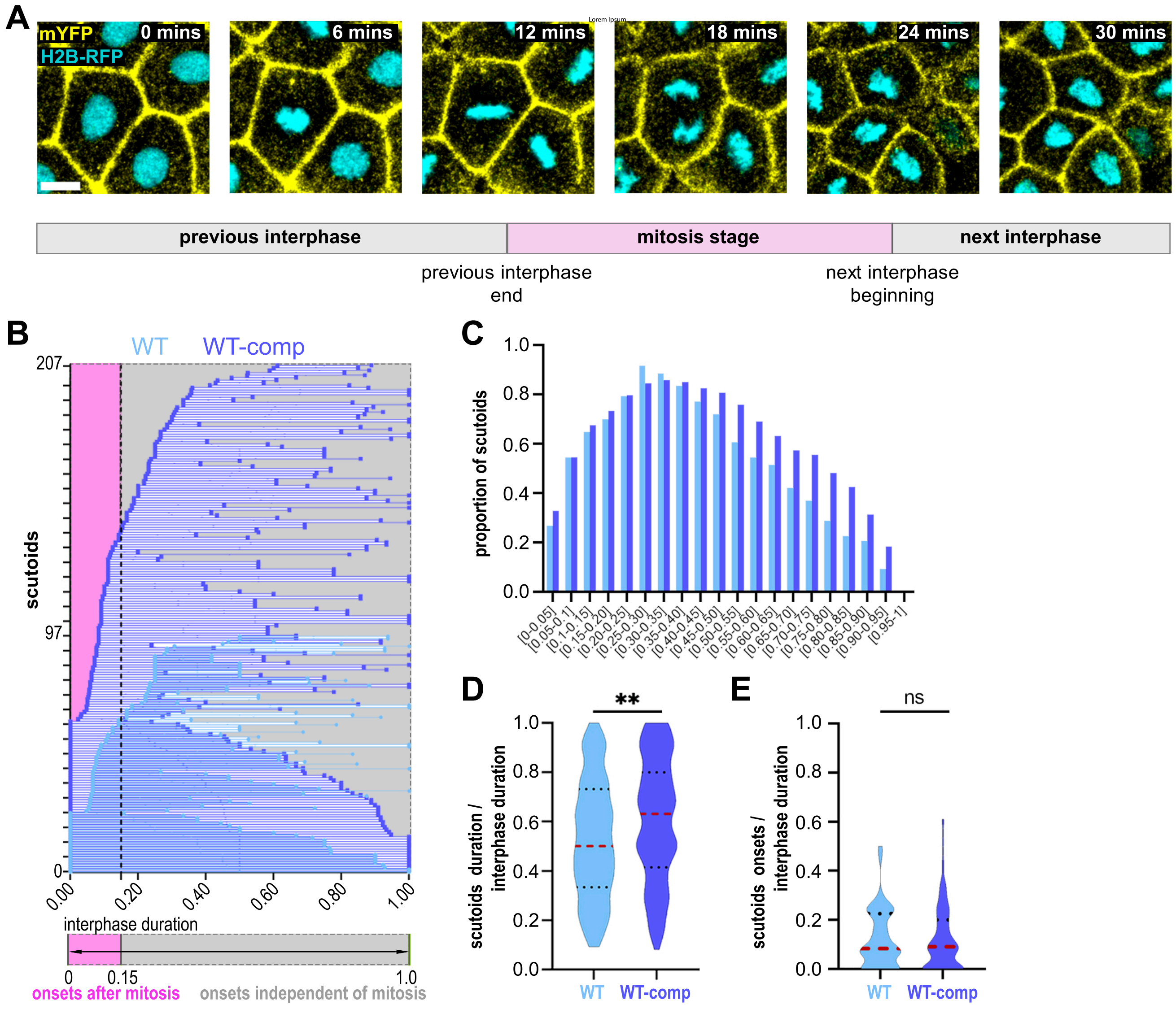

Fig. S7. Tracking of scutoids over time. A) Slices of a representative sea star embryo showing how we establish the beginning and end of an interphase when tracking scutoids. The embryo is expressing the membrane marker mYFP and the nuclei marker H2B-RFP. Panels show the same cells shown in Fig 3C, with additional time points and nuclear marker to highlight cell division. Scale bars, 10 μm. Quantifications of the onsets and end of scutoids individually (**B**), the proportion of scutoids throughout the interphase (**C**), the average scutoids duration (**D**) and the average scutoids onset (**E**). WT: 97 scutoids, 6 embryos, 4 experiments and WT-comp: 207 scutoids, 6 embryos, 5 experiments. Mean (red dotted lines) ± s.d. (black dotted lines). Statistical test: Mann Whitney tests; ns: non-significant; **: p-value <0.01.

Table S1. Summary of statistics. Means and standard deviations for all measurements and conditions included in the manuscripts, and p values for statistical tests performed.

Available for download at
https://journals.biologists.com/dev/article-lookup/doi/10.1242/dev.202362#supplementary-data

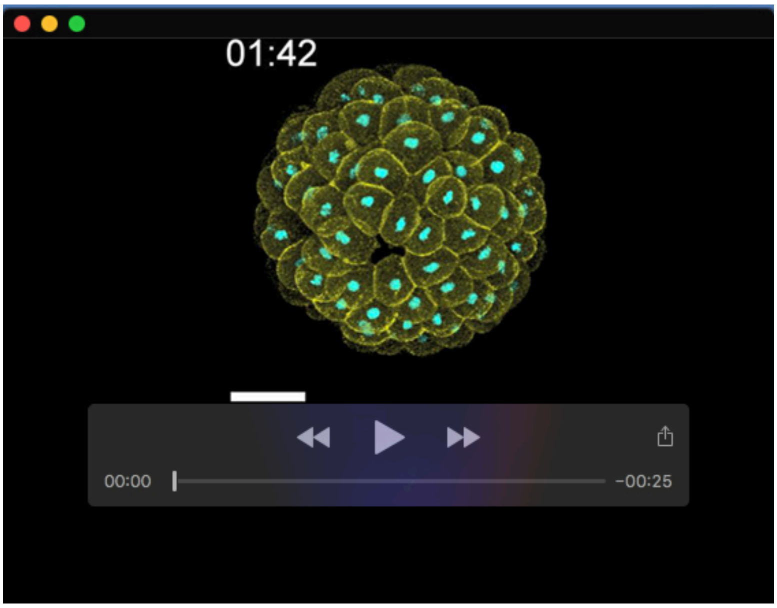

Movie 1. *Patiria miniata* WT embryo, vegetal view. Maximum projection of confocal time-lapse video of a WT embryo expressing a membrane marker (mYFP, yellow) and a nuclear marker (n-CFP, cyan) imaged between the 32- and 2000-cell stages. Vegetal view. Scale bars, 50 µm. Frame interval of 6 minutes, 7 fps.

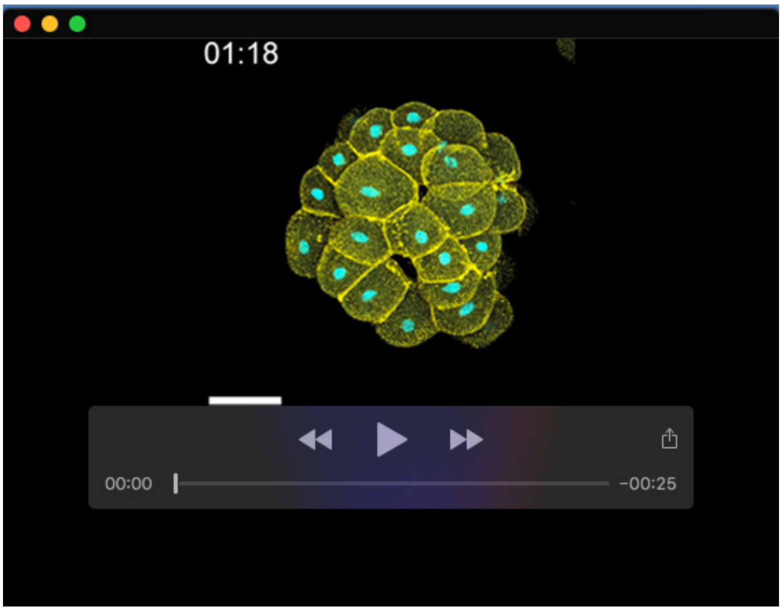

Movie 2. *Patiria miniata* WT-comp embryo, vegetal view. Maximum projection of confocal time-lapse video of a WT-comp embryo expressing a membrane marker (mYFP, yellow) and a nuclear marker (nRFP, cyan) imaged between the 32- and 2000-cell stages. Vegetal view. Scale bars, 50 µm. Frame interval of 6 minutes, 7 fps.

Development • Supplementary information

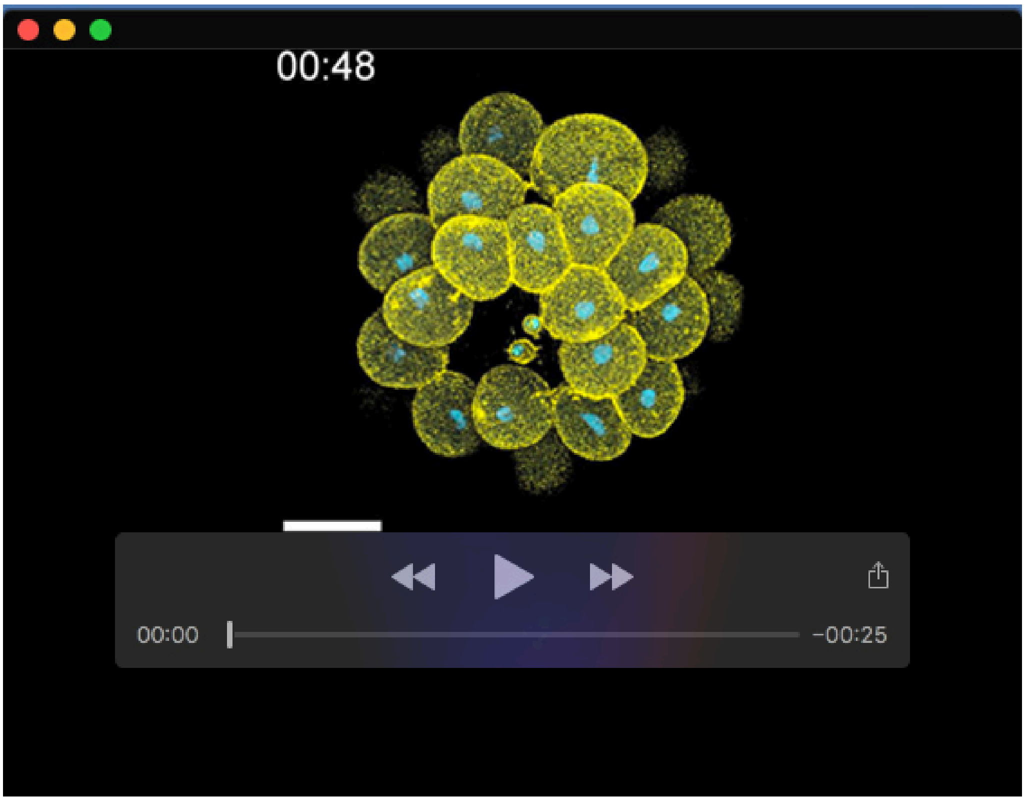

Movie 3. *Patiria miniata* WT embryo, animal view. Maximum projection of confocal time-lapse video of a WT embryo expressing a membrane marker (mYFP, yellow) and a nuclear marker (n-RFP, cyan) imaged between the 32- and 2000-cell stages. Animal view (note the polar bodies). Scale bars, 50 μm. Frame interval of 6 minutes, 7 fps.

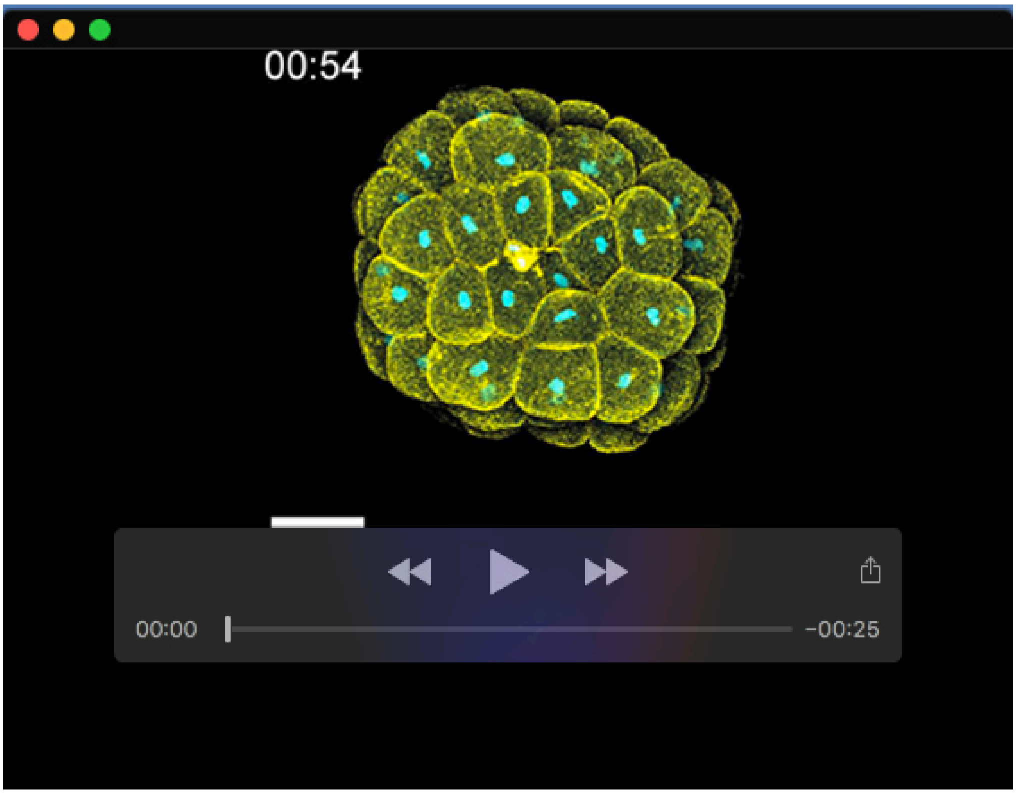

Movie 4. *Patiria miniata* WT-comp embryo, animal view. Maximum projection of confocal time-lapse video of a WT-comp embryo expressing a membrane marker (mYFP, yellow) and a nuclear marker (nRFP, cyan) imaged between the 32- and 2000-cell stages. Animal view (note the polar bodies). Scale bars, 50 μm. Frame interval of 6 minutes, 7 fps.

Development • Supplementary information
