# Supplementary material for: Local and global changes in cell density induce reorganisation of 3D packing in a proliferating epithelium
Source: Development. 2024 May 7;151(20):dev202362. doi: 10.1242/dev.202362 (PMC11112164; doi:10.1242/dev.202362)
Supplement: Supplementary file 1 [file develop-151-202362_review_history.pdf]

Local and global changes in cell density induce reorganisation of 3D packing in a proliferating epithelium

Vanessa Barone, Antonio Tagua, Jesus Á. Andrés-San Román, Amro Hamdoun, Juan Garrido-García, Deirdre C. Lyons and Luis M. Escudero
DOI: 10.1242/dev.202362

Editor: Thomas Lecuit

Review timeline

Original submission:	15 September 2023
Editorial decision:	21 November 2023
First revision received:	6 February 2024
Editorial decision:	25 March 2024
Second revision received:	27 March 2024
Accepted:	28 March 2024

Original submission

First decision letter

MS ID#: DEVELOP/2023/202362

MS TITLE: Local and global changes in cell density induce reorganisation of 3D packing in a proliferating epithelium.

AUTHORS: Vanessa Barone, Antonio Tagua, Jesus Á. Andrés-San Román, Amro Hamdoun, Juan Garrido-García, Deirdre C. Lyons, and Luis M. Escudero

I sincerely apologise for the delay in getting back to you. I have now received all the referees reports on the above manuscript, and have reached a decision. The referees' comments are appended below, or you can access them online: please go to BenchPressand click on the 'Manuscripts with Decisions' queue in the Author Area.

The overall evaluation is clearly positive and we would like to publish a revised manuscript in Development, provided that the referees' comments can be satisfactorily addressed. Please attend to all of the reviewers' comments in your revised manuscript and detail them in your point-by-point response. If you do not agree with any of their criticisms or suggestions explain clearly why this is so. If it would be helpful, you are welcome to contact us to discuss your revision in greater detail. Please send us a point-by-point response indicating your plans for addressing the referees' comments, and we will look over this and provide further guidance.

Reviewer 1

Advance summary and potential significance to field

With the advent of improved image and image analysis tools, our understanding of three-dimensional shape has markedly increased. Scutoids were identified whereby confluent (typically epithelial) cells undergo cell neighbour rearrangements from apical to basal surfaces. Such geometric forms have been identified in a range of systems, from Drosophila to mouse. Yet, most of this analysis has been on static images or limited timelapse movies. Further scutoids were first

© 2024. Published by The Company of Biologists under the terms of the Creative Commons Attribution License (https://creativecommons.org/licenses/by/4.0/).

identified in domains with high curvature. This made it hard to disentangle mechanisms that promoted scutoid formation.

It has previously been observed that scutoids can form in flat tissues, e.g., Gomez et al., eLife 2021. In this case, cell division was posited to be a driver of such rearrangements. Yet, there has been a notable absence of high quality data exploring the spatiotemporal dynamics of scutoid formation and resolution.

Here, Barone et al., tackle this issue with direct spatiotemporal measurements of scutoid formation in the seastar embryo. They quantify the dynamics of scutoid formation at different cell densities. External constraints are applied that alter the scutoid formation process. Finally, they utilise Voronoi models to compare with expected scutoid frequency based on simply geometry. The authors use the results to argue that scutoid formation is an important part of accommodating new cells immediately after division. They further posit that this potentially plays a role in ensuring epithelial tissue integrity during development.

Overall, the paper is well-written and the data largely convincing. They provide a detailed Introduction, which clearly encapsulates the paper aims within the larger literature.

Comments for the author

1. I am generally convinced by the authors claim regarding packing post-mitosis. However, the subsequent proposal that scutoid formation contributes to robustness of the tissue is less well supported. While having n=6 embryos is sufficient for their main conclusions, it is not sufficient to explore robustness. A wider variety of perturbations would be needed to test this claim.
I suggest removing this as a point in the abstract, and restrict the potential robustness to the Discussion. In short, though I suspect the authors are correct, the current evidence doesn't back this claim up as a main result of the manuscript.

2. In a number of the quantifications, the distributions are clearly bimodal – e.g., WT 256 cell in Figure 1E, 128 cell in Figure 2J. In such cases the mean and s.d. are somewhat meaningless. I may be mistaken, but it is not clear if this bimodality was accounted for in the statistical analysis. Relatedly, are there any hallmarks distinguishing these different embryo behaviours? For example, are the embryos so stereotypic that they always have 2n cells at each stage?

3. Data presentation is generally clear. However, in a number of panels the spread on the y-axis is surprisingly large given the data values. This is most apparent in Figure 2G, but also relevant in other panels. The y-axis should be selected to show the spread of points as clearly as possible.

4. Line 196. At this point in the manuscript, the results are largely correlative. It is too bold to say that they "explain" the scutoid formation – these factors are clearly important in scutoid formation, but the current wording implies fully understood.

5. The authors use a 3D Voronoi model to test the role of geometry in scutoid formation. Recent work from the Manning lab has shown that Voronoi models do have limitations in 3D compared with vertex models https://arxiv.org/abs/2306.03987 In particular, one may imagine when there is substantial cell division that the Voronoi model is not as good a representation. Given the relative simple spherical geometry, a 3D vertex model should be checked to make sure that the model predictions are consistent.

6. The results comparing the embryo poles are interesting, with differences in the observed scutoid behaviour. The authors suggest that different material properties may underlie these differences. It should be relatively straightforward to perform laser ablation or AFM to see if there is a clear material difference. If gaining direct measurements is challenging, then force inference methods may be a reasonable way to compare relative material differences between the two poles.

7. Lines 234-236, it is noted that in 5/6 embryos the onset of scutoids with mitosis is very similar. As with point 2 above, are there any differences in the outlier embryos – for example, a different size or was the wave of mitosis more sporadic?
Optional extra: I note that the authors report manually curating the scutoids.

© 2024. Published by The Company of Biologists under the terms of the Creative Commons Attribution License (https://creativecommons.org/licenses/by/4.0/).

Given their large number of events, do they now have sufficient data to train a machine learning approach. This would be very helpful - even if only predictive rather than certain - as identifying scutoids is a laborious process currently.

Reviewer 2

Advance summary and potential significance to field

The authors study the emergence of apico-basal T1 transitions (AB-T1s), which is marked by scutoid-shaped cells, during sea star embryogenesis. They observe correlations between AB-T1s and other properties of cellular packing and cellular dynamics. They conclude that compaction, compression, and cell divisions promote scutoid formation.

AB-T1s are an interesting feature of epihelial cell packing and we are only just beginning to understand them. It has been proposed in the past that anisotropic epithelial curvature can promote their appearance. However, in this manuscript, the authors propose that also other packing properties and dynamical processes can promote their occurrence. In particular, I felt that the transient occurrence of AB-T1s in connection to cell divisions is an important new finding.

Comments for the author

In my view, the reported results are quite interesting and the manuscript is relatively well written. However, I had the impression that there are problems with some of the quantifications and it seems that the interpretation of the results still needs to be clarified more.

In particular:
1. In most of the manuscript the evidence provided are correlations between the occurrence of scutoids and other structural parameters such as cell density/convexity ratio. Overall, I personally found the interpretations of these data, most often phrased in terms of causation, not very convincing. An exception is the compression experiment, but in this case the appearance of more scutoids might also be explained by the higher curvature anisotropy ("surface ratio anisotropy", Fig. 1F).

2. In my view, the most important and striking finding is the correlation of scutoids with the occurrence of cell divisions. But I was confused by the interpretation: the authors combine these experimental results with theoretical predictions to conclude that most of the scutoids can not be explained by static packing properties alone, but are rather the result of dynamic cellular processes. This makes sense, as the data also suggests that many of the scutoids appear only transiently. Yet, in most of the manuscript, the authors seem to interpret their data in a way that static properties like "compaction" or cell density can explain a larger number of scutoids.

3. Some of the quantifications and the modeling are insufficiently explained (see comments below). Also, the "convexity ratio" seems to be a bad proxy for tissue "compaction" (see comments below). Before I can recommend publication, these issues need to be fixed.

Other comments:
4. a) I felt that the manuscript suffers somewhat from the lack of a quantitative definition of tissue "compaction", even though this is proposed as one of the main factors promoting scutoid formation. It seems to refer to a stage of sea star development. Yet, the authors also appear to refer to a geometric cell packing property, when they write, e.g., "the presence of an opening allowed animal cells to remain slightly less compacted for longer" (lines (ls.) 177-178).

The cell number density does not seem to be meant, here, and I suspect that the authors refer to a volume packing fraction (i.e. the fraction of volume that cells fill up as compared to the amount of extracellular fluid between them). Why not stating this explicitly? This would also allow to quantify it directly rather than through some proxies such as the "convexity ratio".

© 2024. Published by The Company of Biologists under the terms of the Creative Commons Attribution License (https://creativecommons.org/licenses/by/4.0/).

b) With a lacking definition of "compaction", statements like "This means a simulation of the tissue with the same surface ratio anisotropy, cell density, and tissue compaction as in the embryos" (ls. 208-209) are confusing.

5. The authors seem to use the "convexity ratio" as one proxy for tissue compaction.
a) The "convexity ratio" is explained in Fig. 1I as "cell volume divided by the convex hull of the cell" (probably meaning "... divided by *the volume of* its convex hull").
b) In the methods, it is defined as "Proportion of the cell volume enclosed by the convex hull of the cell"; yet all of a cell's volume is enclosed by the convex hull of the cell.
c) In what follows, I assume that what is meant is the quotient of the cell volume over the volume of its convex hull. In this case, the convexity ratio of a convex cell is exactly one, and any deviation from one indicates how far away from convexity a cell is.

In lines 143-144, the authors write: "Importantly, given that sea star cells are not protrusive (Movie 1), lower convexity indicates that cells are more tightly packed." I disagree - many tightly / densely packed tissues in 2D and 3D are often made up of perfectly convex cells. (By the way, this is by construction true for usual Voronoi tilings.) Correspondingly, I am not at all convinced that the convexity ratio is a good proxy for cell density or packing fraction. If "compaction" refers to a volume packing fraction, this means that the convexity ratio would be a bad proxy for it.

6. I am not an expert in sea star embryogenesis, but I believe at later stages, there is an invagination at the vegetal pole. Such invaginations may require local anisotropic forces. Could it be that such forces contribute to an increase in the number of scutoids before affecting the overall tissue shape? Could this be an alternative explanation for the larger number of scutoids at the vegetal pole (Fig. 2E)?

7. I think a better description of the Voronoi model used is required, in particular since some details are likely to substantially affect the outcome. I found the methods part too vague about this.
a) Based on the methods part, it seems that what is called "Voronoi model" and "simulation" is rather a geometric construction, so I believe "Voronoi construction" would be a more honest name.
b) lines 467-468: "Referring to 3D Voronoi models established in prior works (Andrés-San Román et al., 2023; Gómez-Gálvez et al., 2018), we simulated ..." It is unclear what "referring to" means here. Do the authors use the same construction as any of these papers?
c) line 472: "For each segmented region, we established the ellipsoidal outer and inner boundaries of the Voronoi." How was the inner and outer boundary ellipsoid established? Was some kind of fit used?
d) lines 473-475: "Then, we calculated the centroid of each segmented cell and used these coordinates as seeds to develop the Voronoi models." It is not clear whether a single 3D Voronoi tiling with these points as seeds was used, or whether a stack of 2D Voronoi tilings for all the layers between the apical and basal surfaces was assembled as in Gómez-Gálvez et al., 2018. If the latter, then what seed points were used in each of those different layers?
e) lines 474-475: "Finally, we implemented the Voronoi algorithm by combining the masks of the segmented regions with the seeds." If the segmentation mask for the region was used to compute the Voronoi tiling, why was it necessary before to explicitly "establish" the inner and outer boundaries before (line 472)?
f) It is not clear what can be learned from a Voronoi construction. The actual cell behavior is almost certainly more complex, including mechanical forces, which would likely lead to a different cell packing. In particular, the number of AB-T1s likely depends quite sensitively on the cell behavior.

8. Some parts of the discussion are confusing. For instance, ls. 304-305: "In this context, we propose that tissue compaction is a proxy for the pressure resisting the lateral expansion of the tissue." It is somewhat unclear what is meant by "pressure resisting the lateral expansion of the tissue". Pressure would rather promote the expansion of the tissue.
typos:
- ls. 233-234: "(5 out 6 WT embryos and 5 out 6 WT-comp embryos)" -> ".. 5 out of 6 .."

<u>Reviewer 3</u>

© 2024. Published by The Company of Biologists under the terms of the Creative Commons Attribution License (https://creativecommons.org/licenses/by/4.0/).

Advance summary and potential significance to field

Barone et al investigate the formation of scutoids (a specific type of geometric solid) during epithelial morphogenesis. Using live imaging of sea star embryos and cutting-edge deep learning-based segmentation, the authors examine the roles of cell density, tissue compaction, and cell proliferation in epithelial architecture. They find that naturally or artificially induced tissue compaction– which is often, but not always, associated with increased cell density– is necessary for scutoid formation. The authors also rule out a role for anisotropic stresses in the apicobasal axis for scutoid formation. The authors then use computational simulations (Voronoi models) without tissue dynamics, such as cell proliferation, to test these experimental results. By comparing simulations and experiments, they find that increased scutoid proportion is linked to cell divisions, particularly after mitosis. The authors propose a role for scutoids in enhancing tissue resilience against compression forces during morphogenesis. I am impressed by their image analysis pipelines, creativity of experiments, and combing computational modeling to test their experimental findings. I enjoyed the unexpected discovery of asynchronous closure of animal and vegetal poles and driving synchronicity by ectopically compressing the embryo. The manuscript overall is well-written, clear, and conceptual.

A major significance of the paper is showing the formation of scutoids in epithelial packing during the compaction of embryos. This is an advance from the previous findings of scutoids in epithelial bending. The causality is, however unclear– 1) Are scutoids driving compaction and thereby making the blastocoel less leaky (a “physiological relevance”), or are they an outcome of the molecular changes driving compaction, likely through changes in surface tension?

Comments for the author

Major comments:
1) The authors may computationally (or experimentally) test their proposition of scutoids, allowing epithelia “to withstand fluctuations in compression forces.”

2) The authors may measure the cell proliferation rate in WT-comp (Figure 1).
Can the increase in cell density be explained exclusively by compaction or weird feedback on the proliferation rate (Lines 160-162)? Especially given the increase in scutoid formation after mitosis.

3) Since volume is conserved in WT-comp, and several lines of evidence correlate compaction, high cell density, and mitosis events with scutoid formation, the authors may investigate the apicobasal axis lengths and individual holoblastic cleavage events to investigate sub-cellular behaviors during individual scutoid formation. Can taller, thinner cells “twist” or form a scutoid more easily? Related, does holoblastic cleavage zipper in a way that is likely to result in a scutoid for longer cells?

4) WT-comp embryos seem to buckle in certain regions (Movie 4). This made me wonder if too much compaction/compression would result in buckling rather than scutoid formation. The author may investigate if scutoids have a higher probability of forming in non-buckled regions and if there is a phase space in which scutoids are more likely than tissue buckling. This can be tested computationally.

Minor comments:
1) Please cite an appropriate review in line 36, for example, Navis, Bagnat 2015

First revision

Author response to reviewers' comments

We sincerely thank all reviewers for the considerate and constructive comments on the

© 2024. Published by The Company of Biologists under the terms of the Creative Commons Attribution License (https://creativecommons.org/licenses/by/4.0/).

manuscript. Point-by-point answers are provided below.

Reviewer 1 Advance Summary and Potential Significance to Field:

With the advent of improved image and image analysis tools, our understanding of three-dimensional shape has markedly increased. Scutoids were identified, whereby confluent (typically epithelial) cells undergo cell neighbour rearrangements from apical to basal surfaces. Such geometric forms have been identified in a range of systems, from Drosophila to mouse. Yet, most of this analysis has been on static images or limited timelapse movies. Further, scutoids were first identified in domains with high curvature. This made it hard to disentangle mechanisms that promoted scutoid formation.

It has previously been observed that scutoids can form in flat tissues, e.g., Gomez et al., eLife 2021. In this case, cell division was posited to be a driver of such rearrangements. Yet, there has been a notable absence of high quality data exploring the spatiotemporal dynamics of scutoid formation and resolution.

Here, Barone et al., tackle this issue with direct spatiotemporal measurements of scutoid formation in the seastar embryo. They quantify the dynamics of scutoid formation at different cell densities. External constraints are applied that alter the scutoid formation process. Finally, they utilise Voronoi models to compare with expected scutoid frequency based on simply geometry. The authors use the results to argue that scutoid formation is an important part of accommodating new cells immediately after division. They further posit that this potentially plays a role in ensuring epithelial tissue integrity during development.

Overall, the paper is well-written and the data largely convincing. They provide a detailed Introduction, which clearly encapsulates the paper aims within the larger literature.

We thank the reviewer for providing such detailed and insightful comments. We are grateful for the positive remarks regarding the clarity of the manuscript and the quality of our data.

Reviewer 1 Comments for the Author:

1. I am generally convinced by the authors claim regarding packing post- mitosis. However, the subsequent proposal that scutoid formation contributes to robustness of the tissue is less well supported. While having n=6 embryos is sufficient for their main conclusions, it is not sufficient to explore robustness. A wider variety of perturbations would be needed to test this claim. I suggest removing this as a point in the abstract, and restrict the potential robustness to the Discussion. In short, though I suspect the authors are correct, the current evidence doesn't back this claim up as a main result of the manuscript.

We agree with the reviewer that the role of scutoid formation in allowing epithelia to better withstand fluctuations in compression forces during morphogenesis is better suited for the Discussion section. We have now removed the statement from the abstract.

2. In a number of the quantifications, the distributions are clearly bimodal – e.g., WT 256 cell in Figure 1E, 128 cell in Figure 2J. In such cases, the mean and s.d. are somewhat meaningless. I may be mistaken, but it is not clear if this bimodality was accounted for in the statistical analysis.

For all statistical tests we have first tested whether the datasets compared are normally distributed and, when not, used an appropriate non-parametric statistical test. We have now included a more detailed description of the statistical tests used in the figure captions (lines 188-191, 249-252, 313-314) and in the methods (lines 649-661).

Relatedly, are there any hallmarks distinguishing these different embryo behaviours? For example, are the embryos so stereotypic that they always have 2n cells at each stage?

The sea star embryos are indeed stereotypic with respect to cell divisions until the 512-cell stage, in the sense that the first 9 cell divisions are synchronous and each cell divides exactly once at each round. As a result, once the round of cell division has ended, the embryo has exactly 2n cells compared to the previous stage. This holds true until the 512-cell stage. After that, i.e. with the

© 2024. Published by The Company of Biologists under the terms of the Creative Commons Attribution License (https://creativecommons.org/licenses/by/4.0/).

10th cell division, cell cycles become more asynchronous, so that the number of cells at following stages is approximately 1000 and 2000, for instance.

While the development of *P. miniata* and this synchronicity of cell divisions is well known, to the best of our knowledge, the data presented here is the first that allows a careful quantification of the timing of cell divisions. As suggested by the reviewers, we have now quantified the relative duration of mitotic waves for the 128-, 256- and 512-cell stages. To this end, we have defined the duration of a mitotic wave as the time elapsed between the moment in which the first cell divides and the moment in which at least 50% of cells have undergone cell division.
This quantification is represented in the **new Fig. S5**. In this representation, relative time 0 is the beginning of the 128-cell stage, i.e. when the first cell of the 64-cell stage embryo divides, and 1 is the end of the 512-cell stage, i.e. when the first cell of the 512-cell embryo divides. Each stage is then split into a “mitotic wave” period (pink), which ends when at least 50% of the cells have divided, and an interphase period (grey), which ends when the first cell generated by the previous round of cell division divides again.

Interestingly, we find some variation in the level of synchronicity (**new Fig. S5A**). To visualize whether this variation may be linked to differences in the timing of scutoid formation and epithelial closure between embryos, we have marked for each embryo the time at which the first scutoid is detected (X) and time in which the epithelium is closed (o). We do not find that the embryos in which scutoids form earlier, or in which the epithelium closes earlier, differ significantly in the synchronicity of their mitotic waves compared to the other embryos. Importantly, we don’t find significant differences between WT and WT-comp embryos with regard to cell division synchronicity (**Fig. S5B**) or overall duration of development (**new Fig. S5C**).

The different embryo behaviors may be attributed to natural variation in the epithelialization process. We believe a much larger dataset would be needed to address the source of such variation, which we think is beyond the scope of this manuscript.

We have now referred to these results in the manuscripts (lines 173-175). We have also updated **Fig. 2B,G, S4B** to include quantifications during the mitotic wave period for each developmental stage.

© 2024. Published by The Company of Biologists under the terms of the Creative Commons Attribution License (https://creativecommons.org/licenses/by/4.0/).

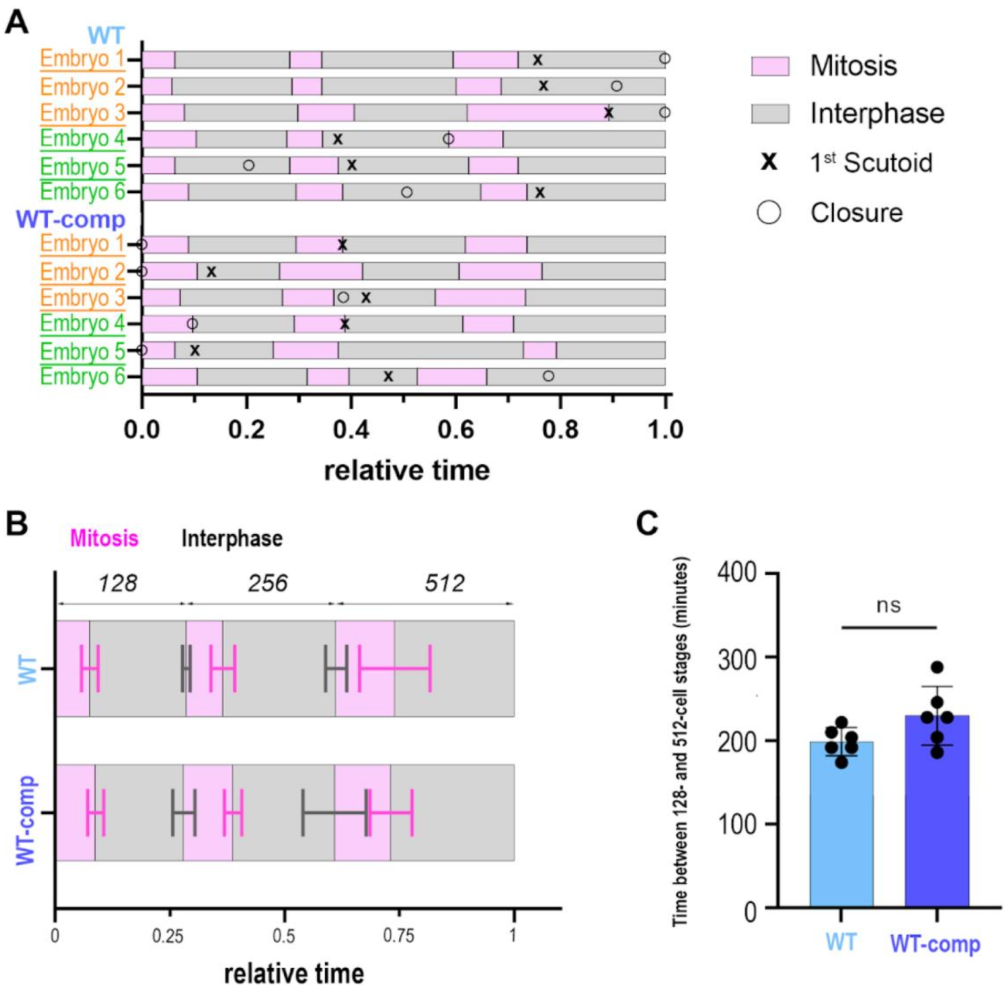

Figure S5. Single-cell tracking of division: analysis of cell proliferation rate per embryo. A) Quantification of mitosis and interphase time intervals per embryo showing the moment the tissue is completely sealed and the first cell adopting scutoidal shape. Quantification of the average time elapsed among each interphase and mitosis intervals (**B**) and between the beginning of the 128-cell stage and the end of 512-cell stage (**C**) in WT and WT-comp embryos. WT: n=150 time points, 6 embryos, 4 experiments. WT-comp: n=150 time points, 6 embryos, 5 experiments. Mean ± s.d. Statistical tests: Student t-test; ns: non-significant.

3. Data presentation is generally clear. However, in a number of panels, the spread on the y-axis is surprisingly large given the data values. This is most apparent in Figure 2G, but also relevant in other panels. The y-axis should be selected to show the spread of points as clearly as possible.

We have now adjusted the range of the y-axis in **Fig. 1C,H, 2E,J, S2B,D,E, S4C-F.** With regard to **Fig. 2G**, the y-axis range was kept consistent with the graph in 2B, to allow a quick appreciation of the differences in epithelial closure between WT (**2B**) and WT-compressed (**2G**). For the same reason, we kept the y-axis range consistent between **2C** and **2H, 2D** and **2I, 2E** and **2J.** We think this important in **Fig. 2**, which will be harder to read if each graph has a different range for the y-axis.

4. Line 196. At this point in the manuscript, the results are largely correlative. It is too bold to say that they "explain" the scutoid formation - these factors are clearly important in scutoid formation, but the current wording implies fully understood.

We have now changed the wording of this sentence to "These experimental results suggested that the dynamics of scutoid formation are *linked to* increase in cell density and tissue compaction."

5. The authors use a 3D Voronoi model to test the role of geometry in scutoid formation. Recent

© 2024. Published by The Company of Biologists under the terms of the Creative Commons Attribution License (https://creativecommons.org/licenses/by/4.0/).

work from the Manning lab has shown that Voronoi models do have limitations in 3D compared with vertex models https://arxiv.org/abs/2306.03987 In particular, one may imagine when there is substantial cell division that the Voronoi model is not as good a representation. Given the relative simple spherical geometry, a 3D vertex model should be checked to make sure that the model predictions are consistent.

We agree with the reviewer that vertex models usually are a very good option to describe tissues in two and three dimensions. In particular, the recent paper from Dr. Manning lab (now published in https://journals.plos.org/ploscompbiol/article?id=10.1371/journal.pcbi.1011724) compares the geometric structure and dynamic sorting behavior in mixtures of two cell types in both 3D vertex and Voronoi models. They show that in this case the vertex model is representing several characteristics of the multilayered tissue that is modeled. *Lawson-Keister et al.*, find that significant differences between vertex and voronoi models arise when simulating unmixing of cells with heterotypic interfacial tensions, "While homogenous tissue seems to behave quite similarly between the two models, …". This is indeed a very specific scenario, important in many biological processes, but not relevant in our case. This is because in our dataset, each 3D voronoi reconstruction encompasses only one cell type (either animal or vegetal) and it is agnostic to interfacial tensions.

Here, we generate specific 3D Voronoi construction for each imaged embryo that starts from the coordinates of the 3D centroids of each cell in all the time-points analyzed. Our aim was, as described now in lines 260-270, that the 3D Voronoi models mimic the region of experimental segmented cells simulating tissues with the same surface ratio anisotropy, cell density, and tissue compaction as observed in the embryos; but, importantly, without the influence of dynamic components such as cell proliferation.

We have successfully used this approach in previous studies (Sánchez-Gutiérrez *et al.*, 2016; Gómez-Gálvez *et al.*, 2018, 2022) to compare the organization of the real epithelium and the voronoi model. In all these cases, we have been able to extract relevant biological conclusions from these experiments. In this work, the voronoi models mimic the monolayer epithelium by using the segmented cellular region as masks. Then, we have distributed the Voronoi seeds based on the 3D centroids of each labelled cell. Our intention was to limit the influence of dynamic processes, such as cell proliferation, but preserving the same surface ratio anisotropy, cell density, and tissue compaction that we observe in the embryos. As a result, we have observed that the frequency of AB-T1s was higher in real tissues than in the models, supporting the notion that dynamic processes drive scutoid formation. Interestingly, similar results were reported in (Gómez-Gálvez *et al.*, 2018) when they compared *Drosophila* egg chambers and Voronoi models mimicking their spheroidal shape (same symmetry axes lengths, cell apico-basal size and number of cells). From stage 1 to 8, the follicular epithelium of the egg chamber undergoes dynamic changes characterized by coordinated migration events and an increase in size mediated by cell proliferation. In this context, the analyses performed under highly dynamic conditions (i.e. stage 4), the proportion of AB-T1s in the real egg chamber was higher than expected considering only the tissue geometry (Gómez-Gálvez *et al.*, 2018). In contrast, under stationary-state conditions (stage 8), the frequency of AB-T1s becomes very similar to the spheroid models.

We have now improved the explanation of the methods used to generate the voronoi models (lines 620-647) and their relevance within the scope of this manuscript (lines 260-270).

6. The results comparing the embryo poles are interesting, with differences in the observed scutoid behaviour. The authors suggest that different material properties may underlie these differences. It should be relatively straightforward to perform laser ablation or AFM to see if there is a clear material difference. If gaining direct measurements is challenging, then force inference methods may be a reasonable way to compare relative material differences between the two poles.

We strongly agree with the reviewer that the comparison between the poles of the embryo is interesting and opens the way for an exciting set of experiments to determine whether the observed differences in scutoid formation are due to differences in the material properties of the animal and vegetal pole. However, we think that these experiments are not entirely straightforward and beyond the scope of this manuscript. Probing tissue stiffness via AFM is

© 2024. Published by The Company of Biologists under the terms of the Creative Commons Attribution License (https://creativecommons.org/licenses/by/4.0/).

challenging, because the envelope will be situated between the AFM bead and the cell/tissue, thereby complicating measurements. Laser ablation may not be effective, because it is better suited to determine whether tissues are under tension or compression more than establishing material properties such as stiffness or viscosity (Sugimura, Lenne and Graner, 2016). We have tried to apply a novel force inference method (Ichbiah *et al.*, 2023) in order to see differences between both poles. However, we have realized that the method cannot work in samples that are confined since the mechanical properties of the embryo are affected by the “envelope” (eggshell of the C. elegans embryo in Ichbiah et al.). We think the method best suited to the task is pipette aspiration assay(Guevorkian and Maître, 2017), as the envelope can be pierced by a relatively small pipette without losing its overall shape and it allows probing cortical tension and viscosity both at the cellular and tissue level (Guevorkian and Maître, 2017). As a matter of fact, VB is in the process of starting her own lab, which will be equipped with a pipette aspiration setup, and plans on performing a thorough spatiotemporal mapping of the physical properties of sea star embryonic cells as one of the first aims in her independent research program. We hope that the reviewer will agree that a careful characterization of material properties of the embryo will find a better home in a follow up manuscript. We have now included these thoughts in the Discussion section (lines 329-394).

7. Lines 234-236, it is noted that in 5/6 embryos the onset of scutoids with mitosis is very similar. As with point 2 above, are there any differences in the outlier embryos – for example, a different size or was the wave of mitosis more sporadic?

According to **Fig. 3F** and the updated lines 287-288, there are 3 of the 12 embryos where less than 60% of scutoids formed right after mitosis. However, we have not found consistent differences in the synchronicity of mitosis (**new Fig. S5**) or in the size of the embryos in which scutoid formation is less strongly associated with cell divisions (**Reviewer Fig. 1),** embryos ids that are not underlined). As discussed in point 2, we believe a much larger dataset would be needed to address the source of such variation, which we think is beyond the scope of this manuscript.

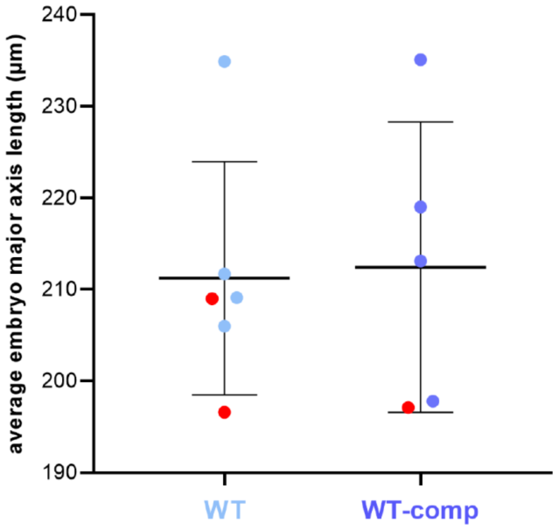

Reviewer Figure 1. Size of embryos in which scutoid formation is less temporally related to cell divisions. The major axis length was measured in each embryo for 10 timepoints during the 512-cell stage. The average value of these measurements is shown for each WT and WT-comp embryo. Embryos where less than 60% of scutoids formed right after mitosis are in red. WT: n= 6 embryos, 4 experiments. WT comp: n=5 embryos, 4 experiments.

Optional extra: I note that the authors report manually curating the scutoids. Given their large number of events, do they now have sufficient data to train a machine learning approach. This would be very helpful - even if only predictive rather than certain - as identifying scutoids is a laborious process currently.

We agree with the reviewer that a machine learning approach to identify what geometric characteristics define a scutoid would be a very valuable tool both for us and the community. We

© 2024. Published by The Company of Biologists under the terms of the Creative Commons Attribution License (https://creativecommons.org/licenses/by/4.0/).

have attempted such an approach with disappointing results so far. Therefore, we have directed our efforts toward improving 3D segmentation, so that automatic detection of scutoid can be possible in complex tissues. We have published recently a software that improves the automatic detection of scutoids (*Andrés-San Román et al. 2023*). In fact, in the segmented images, our code already identifies as scutoids the cells that have different sets of neighbors on their apical and basal sides. However, we have not performed segmentation of all the timepoints of all the movies in this manuscript and so, in some experiments, we tracked them manually, for example to measure for how long a cell maintains the scutoidal shape (**Fig. 3**).

We have now updated the Methods section to clarify the use of automatic detection of scutoids.

******* Reviewer 2 Advance Summary and Potential Significance to Field:**
The authors study the emergence of apico-basal T1 transitions (AB-T1s), which is marked by scutoid-shaped cells, during sea star embryogenesis. They observe correlations between AB-T1s and other properties of cellular packing and cellular dynamics. They conclude that compaction, compression, and cell divisions promote scutoid formation.

AB-T1s are an interesting feature of epihelial cell packing and we are only just beginning to understand them. It has been proposed in the past that anisotropic epithelial curvature can promote their appearance. However, in this manuscript, the authors propose that also other packing properties and dynamical processes can promote their occurrence. In particular, **I felt that the transient occurrence of AB-T1s in connection to cell divisions is an important new finding.**

We thank the reviewer for his/her comments and the positive remarks regarding the importance of the connection between cell divisions and the appearance of AB-T1s.

Reviewer 2 Comments for the Author:
In my view, the reported results are quite interesting and the manuscript is relatively well written. However, I had the impression that there are problems with some of the quantifications and it seems that the interpretation of the results still needs to be clarified more.
In particular:

1. In most of the manuscript the evidence provided are correlations between the occurrence of scutoids and other structural parameters such as cell density/convexity ratio. Overall, I personally found the interpretations of these data, most often phrased in terms of causation, not very convincing. An exception is the compression experiment, but in this case the appearance of more scutoids might also be explained by the higher curvature anisotropy ("surface ratio anisotropy", Fig. 1F).

We have now changed the wording of lines 175-176 to more accurately express the link between cell density/convexity ratio and the appearance of scutoids. In the compression experiment, the dynamics of scutoid formation are not fully explained by changes in curvature anisotropy (**Fig. 1F** and **Fig. S6C-D**).

To further clarify whether the appearance of scutoids in compressed embryos is due to changes in curvature anisotropy, we performed an additional computational experiment as a proof of principle: we used the mask for one WT and one WT-comp embryos and performed a new set of voronoi reconstructions using the average cell density of all our analysed WT and WT-comp. Specifically we modelled a WT embryo mask with either WT or WT-comp cell density and, viceversa, a WT-comp embryo mask with either WT or WT-comp cell density (**Reviewer Fig 2A**). Different from the models in Fig 3A-B, here the seeds were homogeneously distributed to show the contributions of higher curvature anisotropy to the formation of scutoids.

Our findings reveal that in both cases, the Voronoi reconstructions mimicking the WT-comp density show a higher proportion of scutoids compared to their WT density counterparts (**Reviewer Fig. 2**), suggesting that the increased presence of scutoids may be correlated with cell density while curvature anisotropy remains conserved.

© 2024. Published by The Company of Biologists under the terms of the Creative Commons Attribution License (https://creativecommons.org/licenses/by/4.0/).

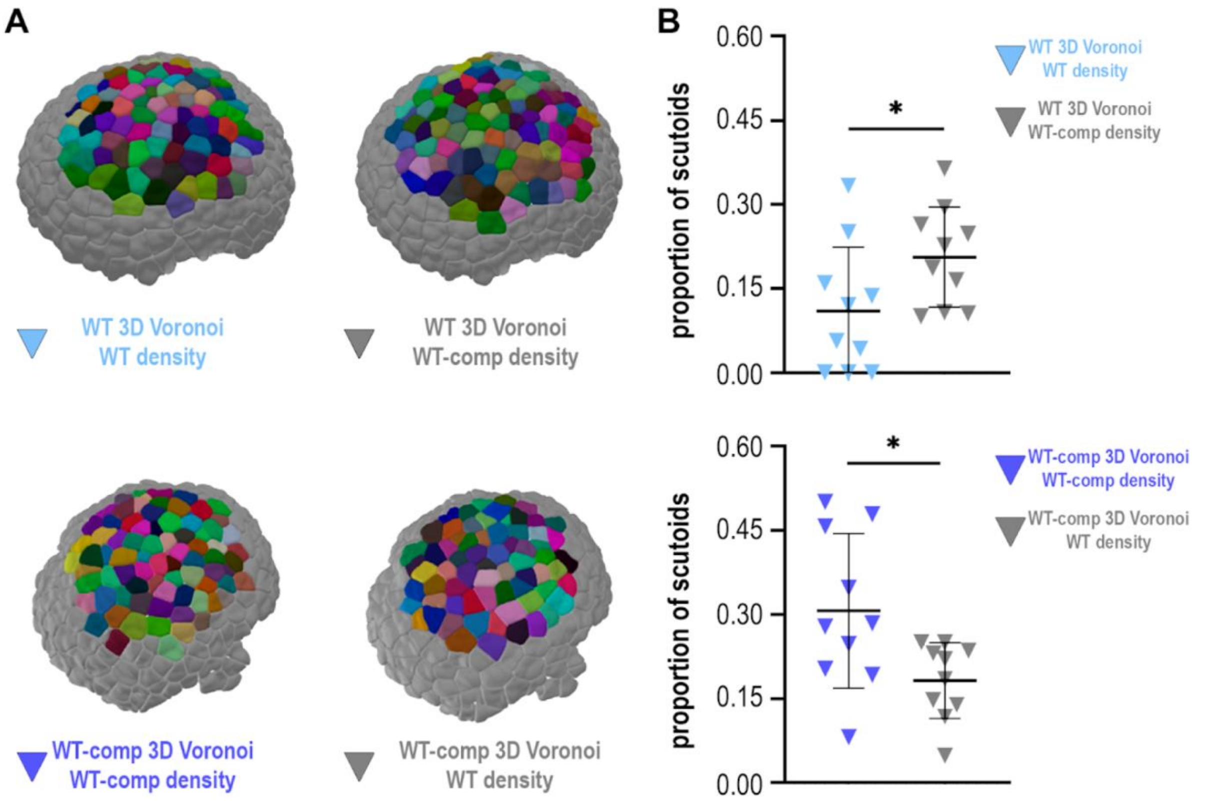

Reviewer Figure 2. Correlation of cell density and 3D packing in 3D Voronoi models. A) Computer rendering of 3D Voronoi models generated from WT and WT-comp embryos masks imitating the same average density as each WT and WT-comp condition at the 512-cell stage. **B)** Quantification of the proportion of scutoids is shown. WT 3D Voronoi with average WT density: n=10 iterations. WT 3D Voronoi with average WT-comp density: n=10 iterations. WT-comp 3D Voronoi with average WT-comp density: n=10 iterations. WT-comp 3D Voronoi with average WT density: n=10 iterations. Mean ± s.d. Statistical test: Student t-tests. *: p-value <0.05.

2. In my view, the most important and striking finding is the correlation of scutoids with the occurrence of cell divisions. But I was confused by the interpretation: the authors combine these experimental results with theoretical predictions to conclude that most of the scutoids can not be explained by static packing properties alone, but are rather the result of dynamic cellular processes. This makes sense, as the data also suggests that many of the scutoids appear only transiently. Yet, in most of the manuscript, the authors seem to interpret their data in a way that static properties like "compaction" or cell density can explain a larger number of scutoids.

We thank the reviewer for pointing out the need for clarification. We interpret our data to show that *changes* in cell density and compaction, which are a consequence of proliferation (cell divisions) in this tissue, are linked to the formation of scutoids. Throughout the manuscript we show how scutoids appear in an embryo that persists in its overall shape but changes in cell density and compaction over time. We use the voronoi models shown in **Fig. 3A** as a tool to ask whether the increase of scutoids during development can be explained solely by the dynamic increment in cell density and compaction. Even though the voronoi models were constructed using the exact geometrical features, number and position of cells of real embryos, so that each virtual cell had the exact same centroid position as the equivalent cell in a real embryo, these models predict lower incidence of scutoids than observed (**Reviewer Fig. 3**). Therefore, we interpret that, aside from the changes in cell density and compaction, another factor must be affecting the induction of scutoids: the cell division.

We have now clarified the dynamic aspect of our interpretation by adding text in lines 260-270.

© 2024. Published by The Company of Biologists under the terms of the Creative Commons Attribution License (https://creativecommons.org/licenses/by/4.0/).

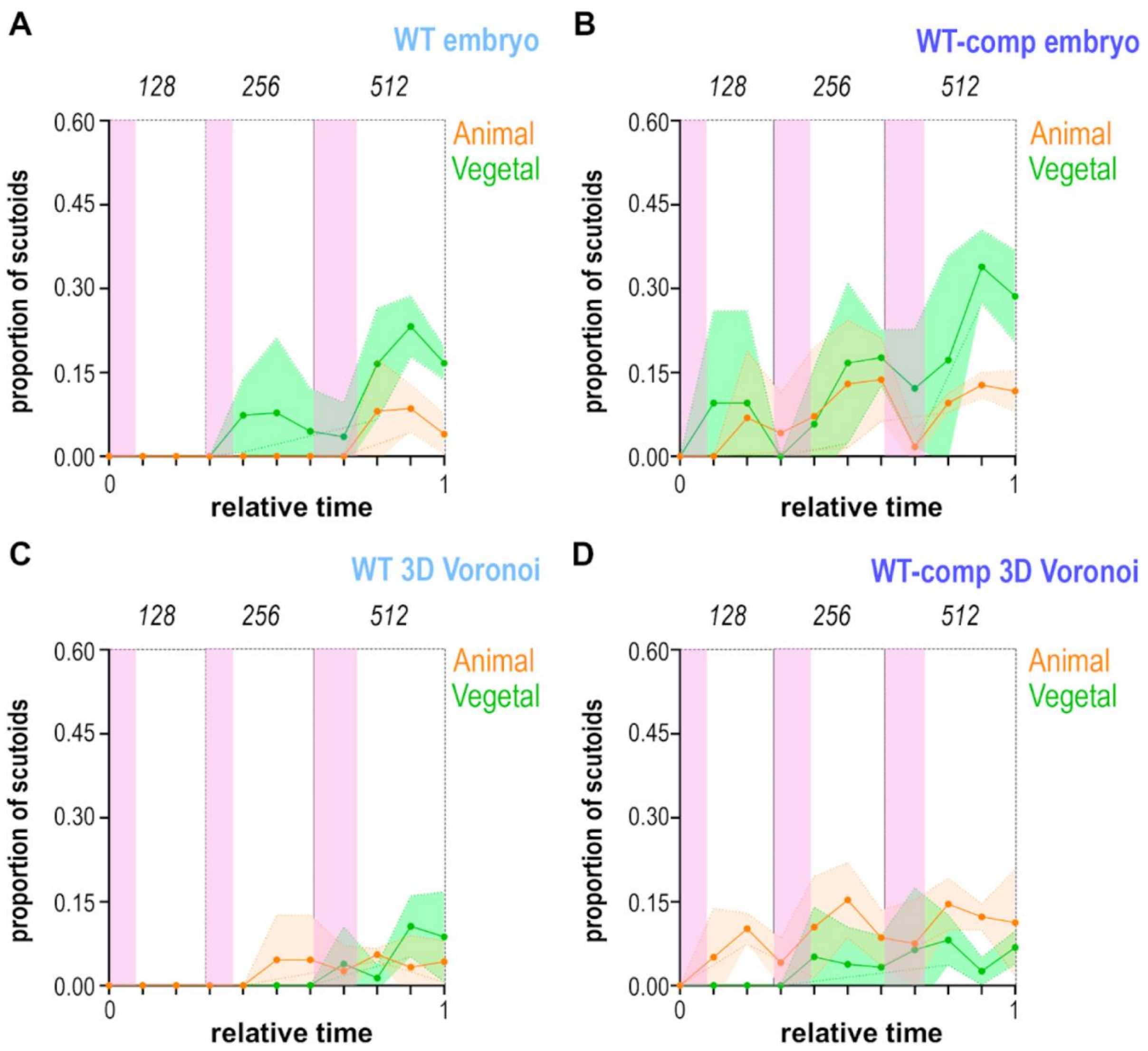

Reviewer Figure 3. Analysis of 3D packing over time. Quantification of the proportion of scutoids is shown in WT (**A**) and WT-comp (**B**) embryos and WT (**C**) and WT-comp (**D**) 3D Voronoi models over time. Relative time 0 corresponds to the first cell division occurring at the 64-cell stage and relative time 1 corresponds to the first cell division occurring at the 512-cell stage. The pink areas denote the average duration of mitotic waves for each stage. WT animal: n= 33, 3 embryos, 3 experiments. WT vegetal: n=33, 3 embryos, 2 experiments. WT-comp animal: n= 33, 3 embryos, 2 experiments. WT-comp vegetal: n=33, 3 embryos, 3 experiments

3. Some of the quantifications and the modeling are insufficiently explained (see comments below). Also, the "convexity ratio" seems to be a bad proxy for tissue "compaction" (see comments below).

We have addressed this point as outlined below, in the answers to points 4, 5 and 7.

Before I can recommend publication, these issues need to be fixed.

Other comments:

4. a) I felt that the manuscript suffers somewhat from the lack of a quantitative definition of tissue "compaction", even though this is proposed as one of the main factors promoting scutoid formation. It seems to refer to a stage of sea star development. Yet, the authors also appear to refer to a geometric cell packing property, when they write, e.g., "the presence of an opening allowed animal cells to remain slightly less compacted for longer" (lines (ls.) 177-178).

© 2024. Published by The Company of Biologists under the terms of the Creative Commons Attribution License (https://creativecommons.org/licenses/by/4.0/).

The cell number density does not seem to be meant, here, and I suspect that the authors refer to a volume packing fraction (i.e. the fraction of volume that cells fill up as compared to the amount of extracellular fluid between them). Why not stating this explicitly? This would also allow to quantify it directly rather than through some proxies such as the "convexity ratio".
b) With a lacking definition of "compaction", statements like "This means a simulation of the tissue with the same surface ratio anisotropy, cell density, and tissue compaction as in the embryos" (ls. 208-209) are confusing.

We thank the reviewer for noting the need for further clarification. The developmental stage at which the epithelium is sealed and cells seem to become more adhesive is commonly referred to as “compaction”. However, we find here that embryo compaction is really a process that takes place over time and via several mechanisms. To improve on clarity, we have removed mention of “embryo compaction” as generally intended in the developmental biology field, and provided a definition of compaction for the purpose of this manuscript (lines 154-156). We have also added a new figure that helps visualize the different aspects of tissue compaction during sea star development (**Fig. 4**) and a new **Fig. S4** to show how convexity relates to compaction. An explanation of how we measure compaction is also provided below, as answer to point 5 raised by reviewer 2.

5. The authors seem to use the "convexity ratio" as one proxy for tissue compaction.
a) The "convexity ratio" is explained in Fig. 1I as "cell volume divided by the convex hull of the cell" (probably meaning "... divided by *the volume of* its convex hull").
b) In the methods, it is defined as "Proportion of the cell volume enclosed by the convex hull of the cell"; yet all of a cell's volume is enclosed by the convex hull of the cell.
c) In what follows, I assume that what is meant is the quotient of the cell volume over the volume of its convex hull.

We are grateful to the reviewer for noticing these imprecisions. We have now corrected the definition of convexity ratio in **Fig. 1I** and in line 583 (**Materials and methods**). Convexity ratio was calculated as the volume of a cell divided by the volume of its convex hull.

In this case, the convexity ratio of a convex cell is exactly one, and any deviation from one indicates how far away from convexity a cell is.
In lines 143-144, the authors write: "Importantly, given that sea star cells are not protrusive (Movie 1), lower convexity indicates that cells are more tightly packed." I disagree - many tightly / densely packed tissues in 2D and 3D are often made up of perfectly convex cells. (By the way, this is by construction true for usual Voronoi tilings.) Correspondingly, I am not at all convinced that the convexity ratio is a good proxy for cell density or packing fraction. If "compaction" refers to a volume packing fraction, this means that the convexity ratio would be a bad proxy for it.

We understand and appreciate the reviewer’s concern that convexity is not a universally valid measure for compaction. For the scope of this manuscript, compaction is used to indicate how tightly cells are packed together. It is perfectly possible for a tissue to become more compact without the cells within it losing convexity. However, during the particular stages of sea star development that we analyze in this manuscript, compaction occurs by several mechanisms:

1) The intracellular space is progressively reduced until all cells are in direct contact with each other along the whole lateral surface. The less intracellular space available the more tightly packed cells are. This is akin to the “volume packing fraction” concept raised by this reviewer and we measure this aspect of compaction as the reduction of ”opening areas” in **Fig 2B,G**.

2) Cell density increases, as measured in **Fig. 1G** and **Fig. 2C,H**. An increase in cell density when cells maintain the same volume is a good measure of compaction. This is the case, for instance, in the comparison between compressed and wt embryos that are at the same developmental stage (**Fig. 1G**). However, when comparing cells between stages, cell density is not necessarily a good measure of changes in how tightly packed the cells are, because cell volumes are halved after each round of cell division.

3) The segmentation of the individual cells of the embryonic epithelium has shown that they bulge into each other, so that the cell-cell contacts are not straight and perpendicular to the

© 2024. Published by The Company of Biologists under the terms of the Creative Commons Attribution License (https://creativecommons.org/licenses/by/4.0/).

epithelial surface, resulting in a loss of convexity (**Reviewer Fig. 4, new Fig. S4 and Fig. 4**). The extent of “lateral bulging” of cells into each other, which results in a reduction of convexity, will be more pronounced the more tightly packed cells are (**new Fig. S4**).

Using these different measures we aimed at describing and account for the different ways compaction occurs in this dynamic tissue. We have now included a clearer definition of compaction as intended in this manuscript (lines 154-156 and 340-346), a **new Fig. S4** showing how lateral bugling and convexity are linked, and an illustration of the different aspects of compaction in **Fig. 4**.

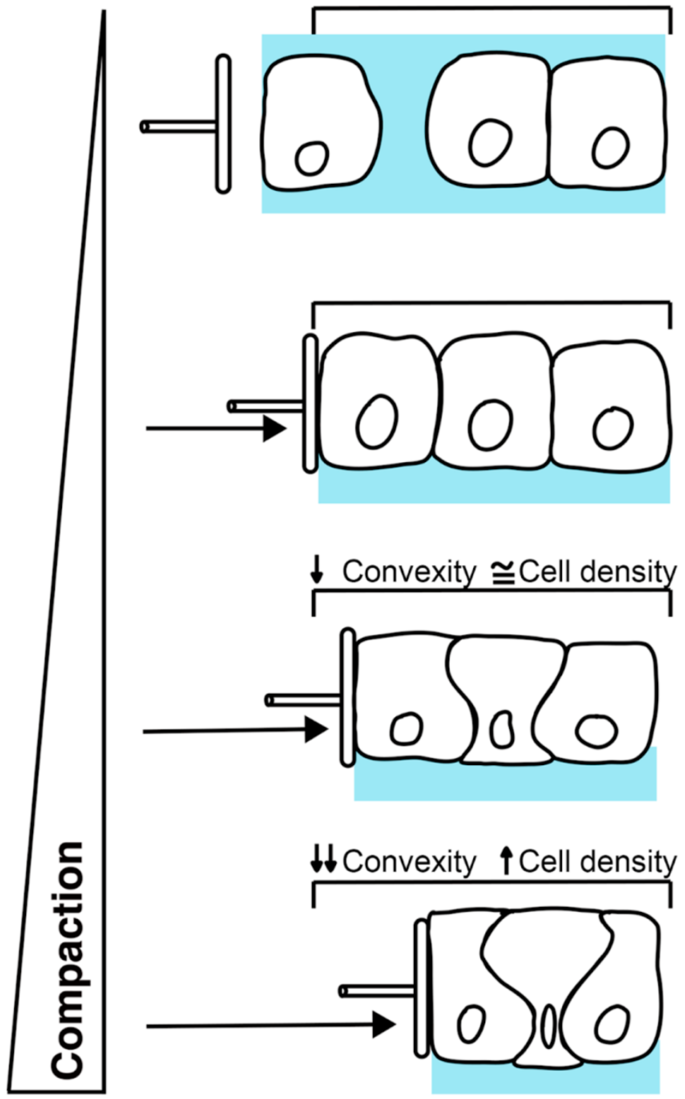

Reviewer Figure 4. Relationship between compaction, cell density, convexity and scutoids during a lateral compression. Schematic representation of compacting tissues. (A) Representation of an idealized tissue under lateral compression, showing how increased compaction may result in several phenomena, including reduction of intercellular space, decreased convexity and increased cell density.

© 2024. Published by The Company of Biologists under the terms of the Creative Commons Attribution License (https://creativecommons.org/licenses/by/4.0/).

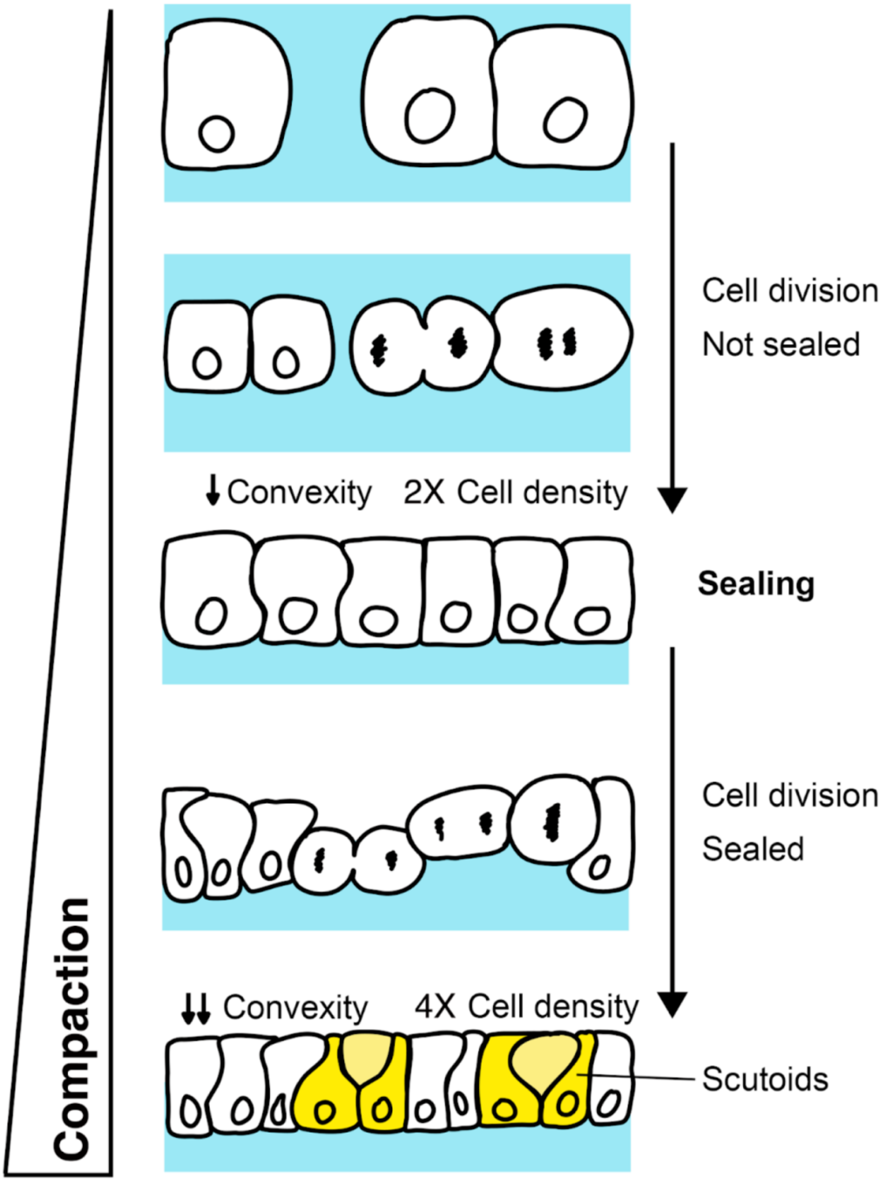

Figure 4. Relationship between compaction, cell density, convexity and scutoids. Schematic representation of compacting tissues. Representation of the process of compaction in sea star embryos, driven by lateral expansion of the epithelium via oriented cell divisions. Initially, cell divisions cause the reduction of interstitial space, until the epithelium is sealed. In this phase, when cells have space to expand laterally, cell divisions do not drive the formation of AB-T1s. Once the epithelium is sealed, and lateral expansion is no longer possible, oriented cell divisions create lateral compression forces that result in lowered convexity and in cells adopting the scutoidal shape.

© 2024. Published by The Company of Biologists under the terms of the Creative Commons Attribution License (https://creativecommons.org/licenses/by/4.0/).

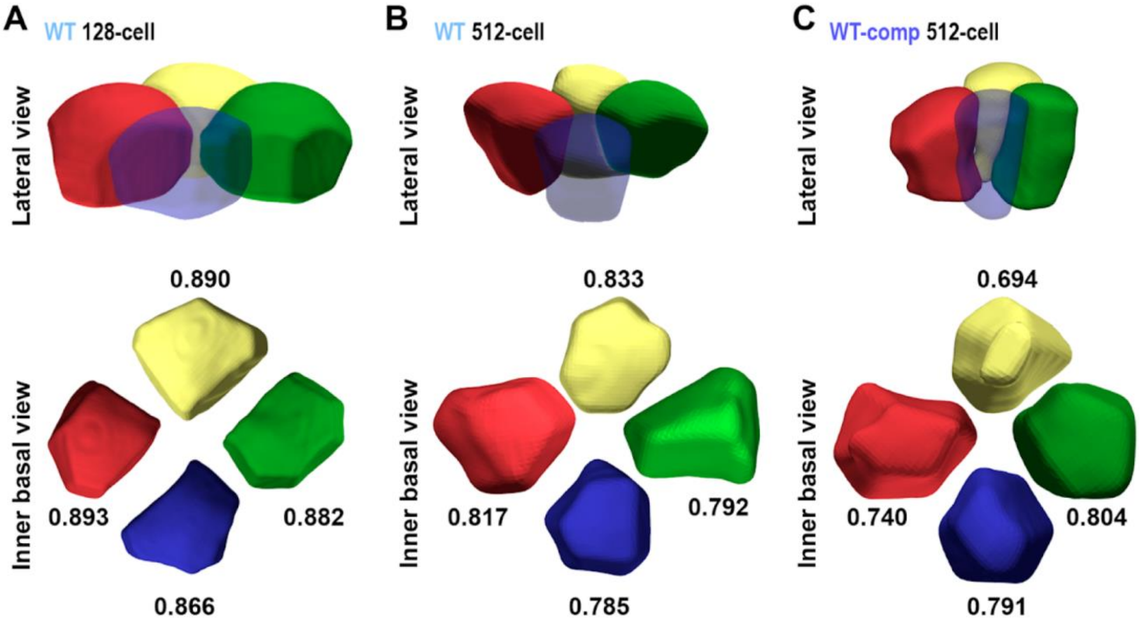

Figure S4. Cell compaction. 3D representation of 4-cell motives from a lateral (Top) and inner (Bottom) view from 128-cell WT embryo (**A**) 512-cell WT embryo (**B**) 512-cell stage (**C**) WT-comp embryo. In the inner view, the numbers displayed next to each cell indicate the individual value of the convexity ratio (**see Materials and methods**).

6. I am not an expert in sea star embryogenesis, but I believe at later stages, there is an invagination at the vegetal pole. Such invaginations may require local anisotropic forces. Could it be that such forces contribute to an increase in the number of scutoids before affecting the overall tissue shape? Could this be an alternative explanation for the larger number of scutoids at the vegetal pole (Fig. 2E)?

The reviewer is correct: invagination at the vegetal pole occurs at later stages of development and marks the beginning of gastrulation. The stages analyzed here are at least 12 hours earlier than the beginning of invagination, as the 512-cell stage occurs approximately at 9 hours post fertilization while invagination begins between 22 and 24 hours post fertilization. The cells undergo many additional rounds of cell division and become strikingly columnar before the epithelium thickens and then invaginates at the vegetal pole. While it is unlikely that anisotropic forces related to invagination are already at play at the stages analyzed here, apical constriction (or expansion) connected to the process of epithelialization might be. If differences in apical constriction between animal and vegetal pole existed, we would expect differences in cell surface ratio (apical surface/ basal surface) or in the dynamics of cell surface ratio. As shown in **Reviewer Fig. 5**, we do not find significant differences in surface ratio between animal and vegetal poles. Therefore, anisotropic forces that may result in surface ratio dynamics do not explain scutoid formation.

© 2024. Published by The Company of Biologists under the terms of the Creative Commons Attribution License (https://creativecommons.org/licenses/by/4.0/).

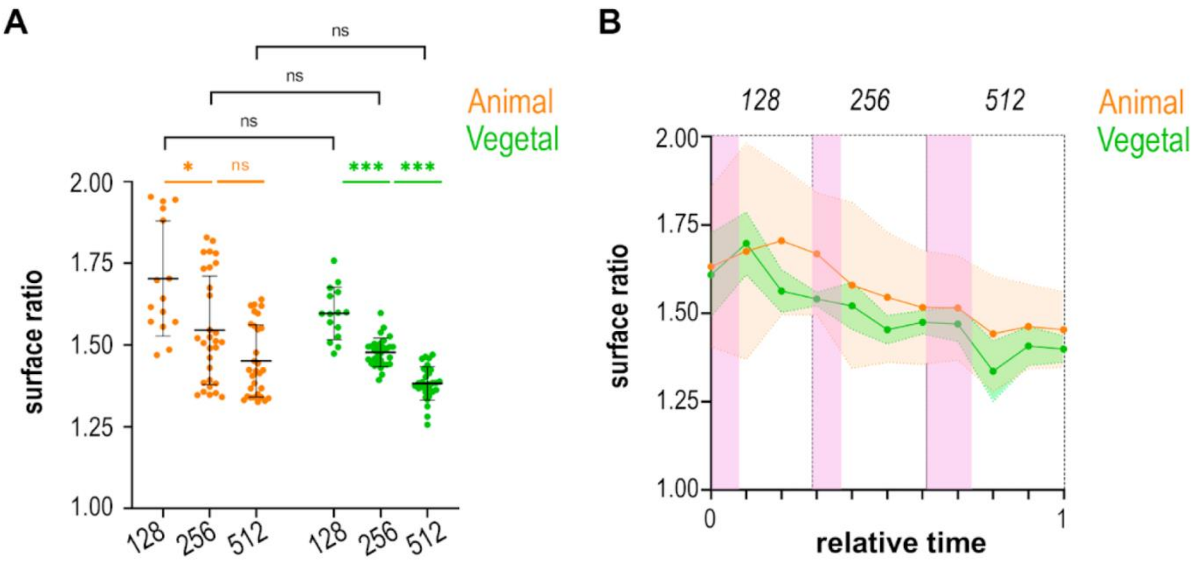

Reviewer Figure 5. Surface ratio dynamics are not linked to scutoid formation. A) Quantification of surface ratio in WT embryos is shown. WT animal: n= 75, 3 embryos, 3 experiments. WT vegetal: n= 75, 3 embryos, 2 experiments. Mean ± s.d. Statistical test: Mann-Whitney tests with Bonferroni multiple comparisons correction (black), Kruskal-Wallis tests with Dunn multiple comparisons correction (orange) and one-way ANOVA test with Tukey multiple comparison correction (green); ns: non-significant; *:p-value <0.05; ***: p-value <0.001. **B)** Comparison of surface ratio in animal and vegetal poles over time. Relative time 0 corresponds to the first cell division occurring at the 64-cell stage and relative time 1 corresponds to the first cell division occurring at the 512-cell stage. The pink areas denote the average duration of mitotic waves for each stage. WT animal: n= 33, 3 embryos, 3 experiments. WT vegetal: n= 33, 3 embryos, 2 experiments.

7. I think a better description of the Voronoi model used is required, in particular since some details are likely to substantially affect the outcome. I found the methods part too vague about this.

a) Based on the methods part, it seems that what is called "Voronoi model" and "simulation" is rather a geometric construction, so I believe "Voronoi construction" would be a more honest name.

We thank the reviewer for this comment. We have realized the need for clarification and we have improved the description of the voronoi model in the Methods section.
Regarding the term “simulation”, we use it to explain that the voronoi model represents some characteristics of the monolayer epithelia (packed cells with apical and basal surfaces). We have followed the advice of the reviewer and changed the text to use the term “construction” making clear that this model mimics the epithelial tissues.

b) lines 467-468: "Referring to 3D Voronoi models established in prior works (Andrés-San Román et al., 2023; Gómez-Gálvez et al., 2018), we simulated ..." It is unclear what "referring to" means here. Do the authors use the same construction as any of these papers?

We are thankful to the reviewer for bringing attention to the need for clarification. We have successfully used this approach in previous studies (*Sánchez-Gutiérrez et al, 2016*)(*Gómez-Gálvez et al., 2018*)(*Gomez-Gálvez et al. 2022*) to compare the organization of the real epithelium and the voronoi model. In all these cases, we have been able to extract relevant biological conclusions from these experiments. In this work we have slightly modified the way that we perform the voronoi model. Specifically, as mentioned in the Methods section (lines 620-647), for the Voronoi constructions, we applied the same 3D Voronoi algorithm described in Andrés-San Román et al., 2023. The space, determined by the experimental segmented region, was filled using the 3D centroids of each cell as seeds. Consequently, the unoccupied space was filled by the nearest individual cells (Voronoi seeds).

© 2024. Published by The Company of Biologists under the terms of the Creative Commons Attribution License (https://creativecommons.org/licenses/by/4.0/).

c) line 472: "For each segmented region, we established the ellipsoidal outer and inner boundaries of the Voronoi." How was the inner and outer boundary ellipsoid established? Was some kind of fit used?

We thank the reviewer for pointing out the need for clarification. These sentences aim to emphasize that the Voronoi constructions exhibit similar inner and outer boundaries as the real tissues. This similarity arises because we utilized the mask of the segmented region in computing the Voronoi algorithm. Additionally, after the generation of the Voronoi construction, we extract the outer and inner layers, facilitating the identification of neighbor exchanges between both surfaces (cells adopting scutoid conformations). We have changed the Methods section accordingly.

d) lines 473-475: "Then, we calculated the centroid of each segmented cell and used these coordinates as seeds to develop the Voronoi models." It is not clear whether a single 3D Voronoi tiling with these points as seeds was used, or whether a stack of 2D Voronoi tilings for all the layers between the apical and basal surfaces was assembled as in Gómez-Gálvez et al., 2018. If the latter, then what seed points were used in each of those different layers

We appreciate the reviewer's observation regarding the requirement for clarification. A single 3D Voronoi tiling with the coordinates of the segmented cellular centroids as seeds was used. Then, we applied a Voronoi algorithm filling the unoccupied space of the bounded region by the nearest Voronoi seeds. We have changed the Methods section accordingly.

e) lines 474-475: "Finally, we implemented the Voronoi algorithm by combining the masks of the segmented regions with the seeds." If the segmentation mask for the region was used to compute the Voronoi tiling, why was it necessary before to explicitly "establish" the inner and outer boundaries before (line 472)?

The reviewer is correct; it is not necessary, and we did not perform that action before the generation of the Voronoi constructions. In line 472, our intention was to clarify that real and simulated tissues share similar inner and outer boundaries. Following the generation of Voronoi constructions, we defined the outer and inner layers of the simulated tissues to identify the neighbors of each Voronoi cell at the outer and inner surfaces. This process enabled us to detect exchanges between the two regions and quantify the proportion of scutoids in the Voronoi constructions. We have changed the Methods section accordingly.

f) It is not clear what can be learned from a Voronoi construction. The actual cell behavior is almost certainly more complex, including mechanical forces, which would likely lead to a different cell packing. In particular, the number of AB-T1s likely depends quite sensitively on the cell behavior.

We thank the reviewer for pointing out the need for clarification. We agree with the reviewer that the Voronoi model fails to capture the role of dynamic cell behaviours and of mechanical forces in the occurrence of AB-T1s. We use the Voronoi models shown in **Fig. 3A** as a tool to ask whether the increase of scutoids during development can be explained solely by the combination of the geometry and the dynamic increment in cell density and compaction. Even though the Voronoi models were constructed using the exact geometrical features, number and position of cells of real embryos, (so that each virtual cell had the exact same centroid position as the equivalent cell in a real embryo), these models predict lower incidence of scutoids than observed (**Reviewer Fig. 3**). Therefore, we conclude that the results from the Voronoi model suggest that other cell behaviours, such as cell division, may induce the formation of AB-T1s.

We have now improved the explanation for the scope of the Voronoi models (lines 260-270).

8. Some parts of the discussion are confusing. For instance, ls. 304-305: "In this context, we propose that tissue compaction is a proxy for the pressure resisting the lateral expansion of the tissue." It is somewhat unclear what is meant by "pressure resisting the lateral expansion of the tissue". Pressure would rather promote the expansion of the tissue.

We have now clarified how oriented cell divisions cause the tissue to thin and expand laterally

© 2024. Published by The Company of Biologists under the terms of the Creative Commons Attribution License (https://creativecommons.org/licenses/by/4.0/).

within the confines of the fertilization envelope, and how this process results in progressive compaction (**Fig 4**, lines 340-346). We have also changed this sentence to: “In this context, we propose that tissue compaction, measured here as reduced cell convexity, is a proxy for the pressure acting between cells within a tightly packed proliferating epithelium”.

typos:
- ls. 233-234: "(5 out 6 WT embryos and 5 out 6 WT-comp embryos)" -> ".. 5 out of 6 .."
We have now corrected these typos.

******* Reviewer 3 Advance Summary and Potential Significance to Field:**
Barone et al investigate the formation of scutoids (a specific type of geometric solid) during epithelial morphogenesis. Using live imaging of sea star embryos and cutting-edge deep learning-based segmentation, the authors examine the roles of cell density, tissue compaction, and cell proliferation in epithelial architecture. They find that naturally or artificially induced tissue compaction–which is often, but not always, associated with increased cell density– is necessary for scutoid formation. The authors also rule out a role for anisotropic stresses in the apicobasal axis for scutoid formation. The authors then use computational simulations (Voronoi models) without tissue dynamics, such as cell proliferation, to test these experimental results. By comparing simulations and experiments, they find that increased scutoid proportion is linked to cell divisions, particularly after mitosis. The authors propose a role for scutoids in enhancing tissue resilience against compression forces during morphogenesis. I am impressed by their image analysis pipelines, creativity of experiments, and combing computational modeling to test their experimental findings. I enjoyed the unexpected discovery of asynchronous closure of animal and vegetal poles and driving synchronicity by ectopically compressing the embryo. The manuscript overall is well-written, clear, and conceptual.

A major significance of the paper is showing the formation of scutoids in epithelial packing during the compaction of embryos. This is an advance from the previous findings of scutoids in epithelial bending. The causality is, however, unclear- 1)Are scutoids driving compaction and thereby making the blastocoel less leaky (a “physiological relevance”), or are they an outcome of the molecular changes driving compaction, likely through changes in surface tension?

We thank the reviewer for the careful consideration of our manuscript. We are grateful for the positive remarks regarding the clarity of the manuscript.

Our results show that the formation of scutoid requires cells to be tightly packed, but scutoids do not drive compaction *per se*. Our results suggest that the increase of the proportion of scutoids is linked to the increase of cell density (global) or cell divisions (local). These two factors affect the pressure between the epithelial cells within the confined embryo. Moreover, the finding that cells acquiring the scutoidal shape do so shortly after cell division and that AB-T1s transitions are then resolved over time, while the tissue maintains its level of compaction, further points at the formation of scutoids being a consequence of the transient increase of local pressure after cell division. We suggest that, in turn, the fact that a proportion of cells acquires the scutoidal shape may confer higher resistance to compression, purely based on a structural principle (Dhari and Patel, 2022). We agree with the reviewer that this suggestion is speculative at this point, and we have therefore removed it from the abstract.
As suggested here by this reviewer, the molecular mechanisms underlying AB-T1s, and their effects on tissue integrity, are currently unclear. We think that the present manuscript paves the way for further studies addressing these points.

Reviewer 3 Comments for the Author:

Major comments:

1) The authors may computationally (or experimentally) test their proposition of scutoids, allowing epithelia “to withstand fluctuations in compression forces.”

We agree with the reviewer that the role of scutoid formation in allowing epithelia to better withstand fluctuations in compression forces requires further experiments to be clarified. We have

© 2024. Published by The Company of Biologists under the terms of the Creative Commons Attribution License (https://creativecommons.org/licenses/by/4.0/).

now removed the statement from the abstract and left it as an outlook point in the Discussion section.

2) The authors may measure the cell proliferation rate in WT-comp (Figure 1). Can the increase in cell density be explained exclusively by compaction or weird feedback on the proliferation rate (Lines 160-162)? Especially given the increase in scutoid formation after mitosis.

We have now quantified the proliferation rate in each embryo, expressed as synchronicity of mitotic waves. It is important to note that, in both WT and WT-comp, we have defined the stages of development analyzed based on the rounds of cell division, not based on developmental time. This approach allows us to meaningfully compare embryos that may develop at slightly different speeds. One source of variation in cell density can then be in how synchronous each round of cell division is in different embryos. Therefore, we have now quantified the relative duration of mitotic waves for the 128-, 256- and 512-cell stages. To this end, we have defined the duration of a mitotic wave as the time elapsed between the moment in which the first cell divides and the moment in which at least 50% of cells have undergone cell division.
This quantification is represented in **new Fig. S5A-B**. In this representation, relative time 0 is the beginning of the 128-cell stage, i.e. when the first cell of the 64-cell stage embryo divides, and 1 is the end of the 512-cell stage, i.e. when the first cell of the 512-cell embryo divides. Each stage is then split into a "mitotic wave" period (pink), which ends when at least 50% of the cells have divided, and an interphase period (gray), which ends when the first cell generated by the previous round of cell division divides again.
As shown in **new Fig. S5B-C**, we find no significant difference in the synchronicity of mitotic waves nor in the overall duration of development between WT and WT-comp embryos.

We have now added relevant text in lines 173-176.

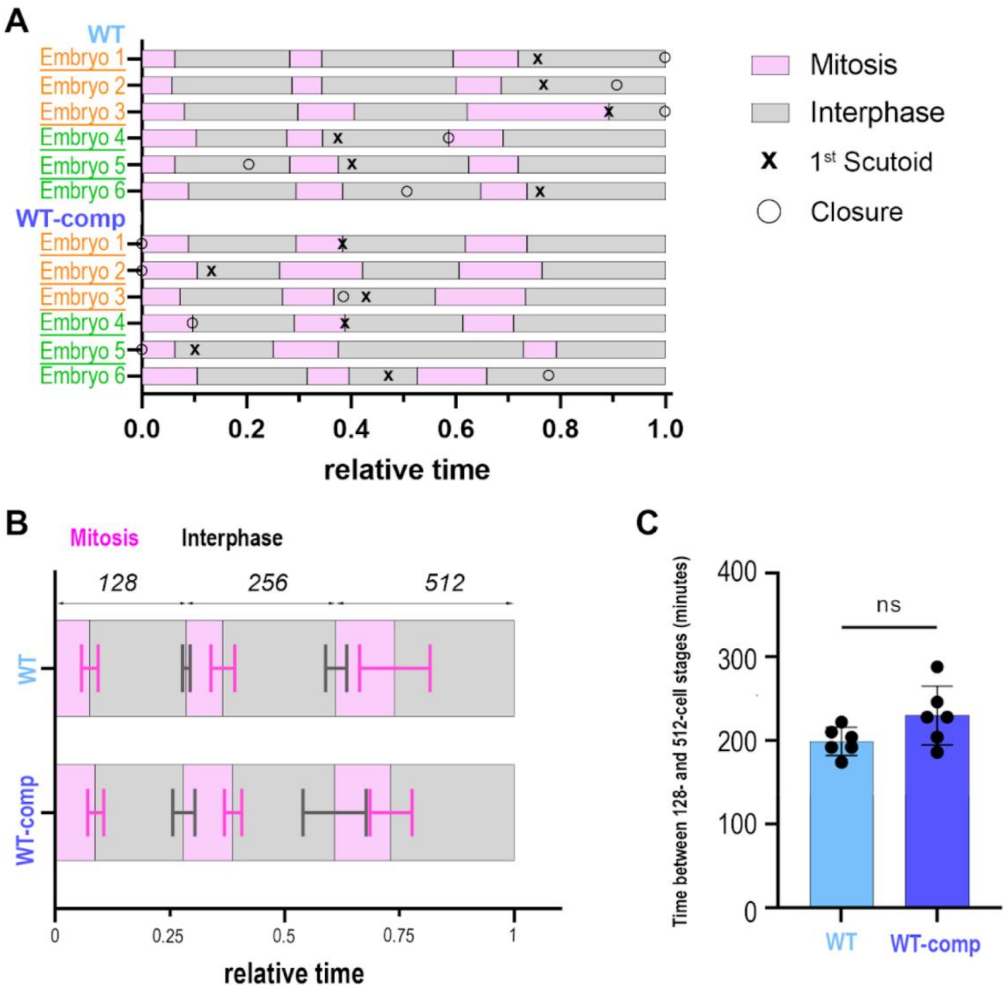

© 2024. Published by The Company of Biologists under the terms of the Creative Commons Attribution License (https://creativecommons.org/licenses/by/4.0/).

Figure S5. Single-cell tracking of division: analysis of cell proliferation rate per embryo. A) Quantification of mitosis and interphase time intervals per embryo showing the moment the tissue is completely sealed and the first cell adopting scutoidal shape. Quantification of the average time elapsed among each interphase and mitosis intervals (**B**) and between the beginning of the 128-cell stage and the end of 512-cell stage (**C**) in WT and WT-comp embryos. WT: n=150 time points, 6 embryos, 4 experiments. WT-comp: n=150 time points, 6 embryos, 5 experiments. Mean ± s.d. Statistical tests: Student t-test; ns: non-significant.

3) Since volume is conserved in WT-comp, and several lines of evidence correlate compaction, high cell density, and mitosis events with scutoid formation, the authors may investigate the apicobasal axis lengths and individual holoblastic cleavage events to investigate sub-cellular behaviors during individual scutoid formation. Can taller, thinner cells "twist" or form a scutoid more easily? Related, does holoblastic cleavage zipper in a way that is likely to result in a scutoid for longer cells?

We have now compared the volume and apico-basal axis length (cell height) of cells with scutoid and frustum shapes (**Reviewer Fig. 6**). In all cases the values for scutoid shaped cells are within the spread of values for non-scutoidal cells. We find that in WT there is no difference in the average volume and cell height between scutoidal and frustum shaped cells at the 512-cell stage. At the 256-cell stage, the cells that adopt the scutoidal shape have higher average volume, but this difference does not translate into a difference in cell height. In WT-comp embryos, instead, cells that adopt the scutoidal shape have both higher average volume and average cell heights than their frustum shaped counterparts. These results can be interpreted in two ways

1. AB-T1s are more likely to occur between taller cells, although the apico-basal axis length is not a strong predictor of which cells will become scutoids.
2. Larger cells in a tissue experience higher levels of compression, which results in increased cell height, and are, therefore, more likely to establish AB-T1s.

The fact that the correlation between cell volume, cell height and occurrence of scutoids is more strongly observed in compressed embryos, favors the second hypothesis. However, a deeper understanding of the molecular mechanisms underlying AB-T1s is needed to determine whether apico-basal axis length *per se*, may influence scutoid formation.

© 2024. Published by The Company of Biologists under the terms of the Creative Commons Attribution License (https://creativecommons.org/licenses/by/4.0/).

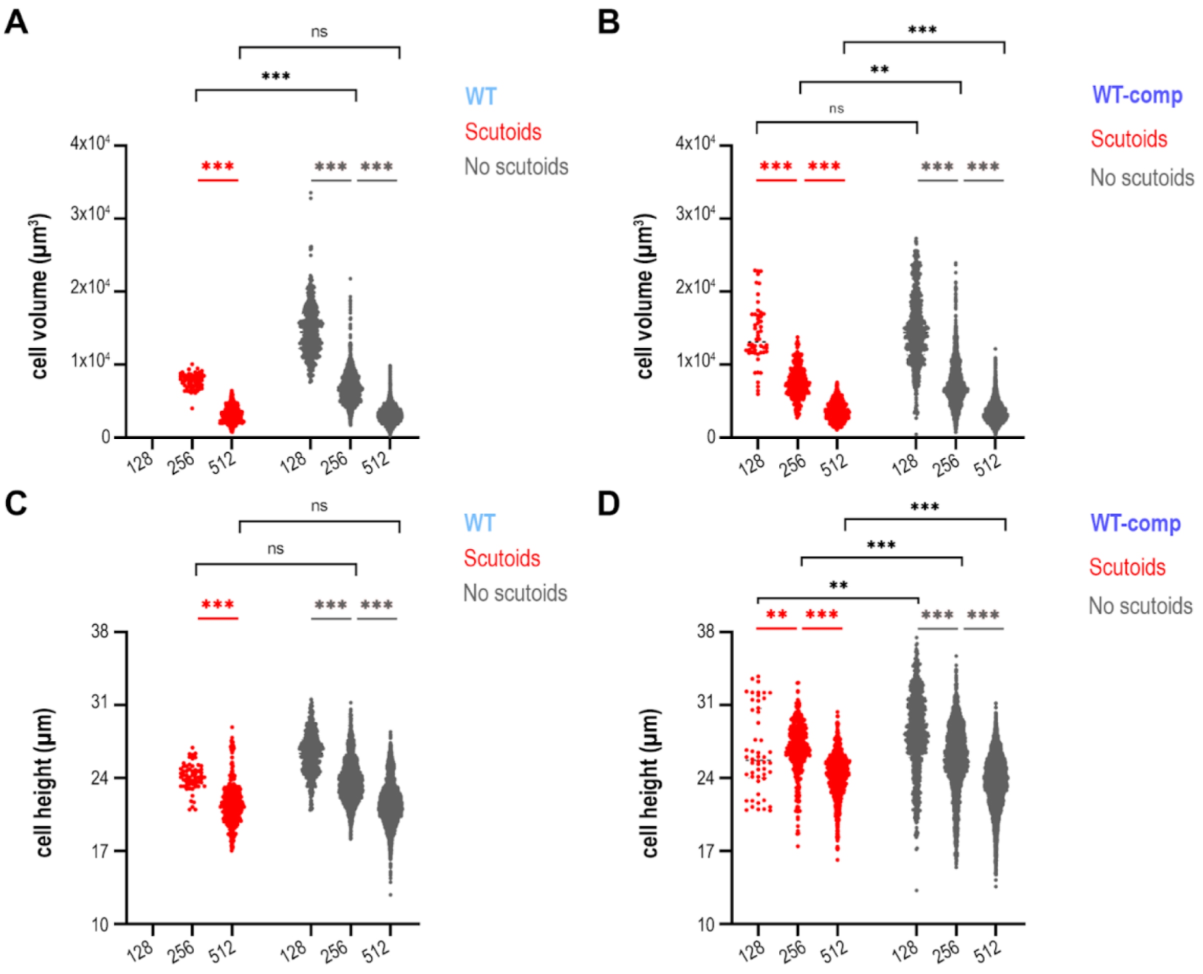

Reviewer Figure 6. Local comparison between cells adopting scutoid and frustum conformations. Quantification of cell volumes (**A-B**), and cell heights (**C-D**) in WT and WT-comp embryos between cells acquiring a scutoidal shape or a frustum shape. WT: 604 scutoids and 6929 frusta in 150 timepoints, 6 embryos, 4 experiments and WT-comp: 1436 scutoids, 8509 frusta in 150 timepoints, 6 embryos, 5 experiments. Mean ± s.d. Statistical tests: Mann-Whitney tests with Bonferroni multiple comparisons correction (black) and Kruskal-Wallis tests with Dunn multiple comparisons correction (red and grey); ns: non-significant; *: p-value <0.05; **: p-value <0.01; ***: p-value <0.001.

4) WT-comp embryos seem to buckle in certain regions (Movie 4). This made me wonder if too much compaction/compression would result in buckling rather than scutoid formation. The author may investigate if scutoids have a higher probability of forming in non-buckled regions and if there is a phase space in which scutoids are more likely than tissue buckling. This can be tested computationally.

We agree with the reviewer that the relationship between tissue compression, scutoid and buckling is an interesting one. As mentioned in the Methods section (lines 573-574), we selected a valid region formed by the cells whose centroid lay within 30 µm from the embryo surface closest to the objective. This delimitation of regions is represented in **Reviewer Fig. 7A.** While all compressed embryos buckle in some regions (**Reviewer Fig. 7A**), in 4 out of 6 embryos a buckling region falls in the portion of the tissue that we analyzed (**Reviewer Fig. 7B**). To study whether scutoids appear or not in buckled regions, we visualized their spatial distribution and found that scutoids can appear in areas where the tissue buckles (**Reviewer Fig. 7C**). Observing our data, we recognize that there may be a bias by which scutoids are more frequent in non-buckled regions at the 512-cell stage (see embryos 5 and 6 in **Reviewer Fig 7C**). However, our sample size is too small to determine whether a significant difference exists. Moreover, in embryo 1, where the buckling is most evident, we were unable to faithfully segment a proportion of cells involved in the buckling, limiting the power of a spatial analysis (**Reviewer Fig. 7B,C,** also see **Material and**

© 2024. Published by The Company of Biologists under the terms of the Creative Commons Attribution License (https://creativecommons.org/licenses/by/4.0/).

Methods). It is important to note that none of the WT embryos showed any buckling. Therefore the main result of this experiment, showing that compression induces AB-T1s is valid, even though it is possible that even more scutoids may have formed if embryos were compressed in a way that did not result in buckling.

Building a theoretical model accounting for the role of compression and buckling will require new complex computational models and we are excited to work on this aspect of epithelial morphogenesis in the future. We hope that the reviewer will agree that these experiments are better suited for a follow up manuscript.

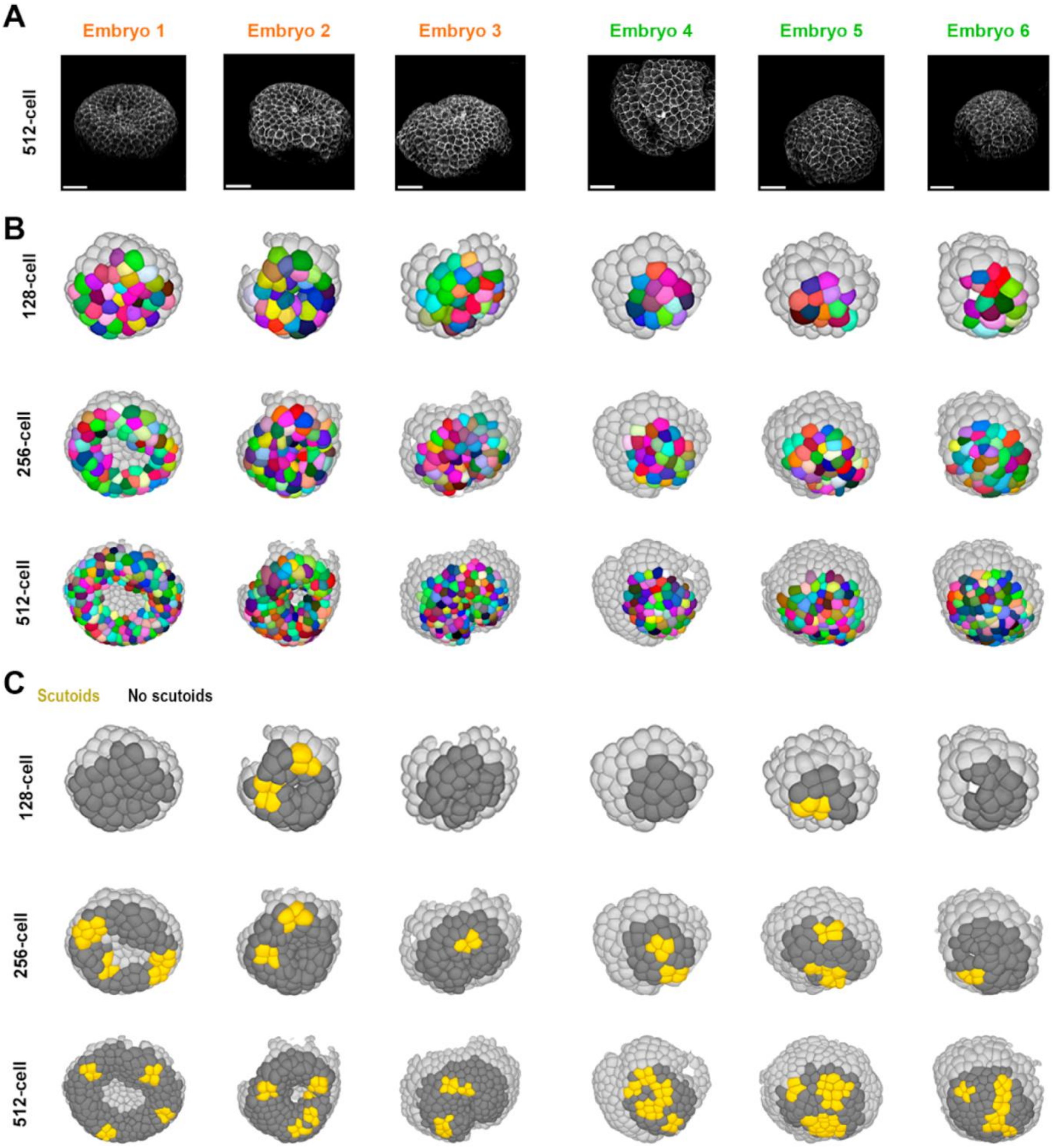

Reviewer Figure 7. Cartography of WT-comp embryos and scutoids patterns analysis. A) Maximum projections of each sea star embryo analyzed. Scale bars, 50 µm. We can observe different buckling events from embryos 1 to 4. **B)** Computer rendering of each segmented sea star embryo at 128-,256- and 512-cell stage representing the colored valid region and the grey invalid area from a frontal perspective. **C)** Computer rendering of each segmented sea star embryo at

© 2024. Published by The Company of Biologists under the terms of the Creative Commons Attribution License (https://creativecommons.org/licenses/by/4.0/).

128-,256- and 512-cell stage representing the cells that adopt a scutoid (yellow) and frusta (black) conformations in a specific timepoint.

Minor comments:

1) Please cite an appropriate review in line 36, for example, Navis, Bagnat 2015

We have now added the suggested reference (Navis and Bagnat, 2015) (line 43).

References

Dhari, R.S. and Patel, N.P. (2022) 'On the crushing behaviour of scutoid-based bioinspired cellular structures', *International Journal of Crashworthiness*, 27(4), pp. 945–954. Available at: https://doi.org/10.1080/13588265.2020.1866859.

Gómez-Gálvez, P. *et al.* (2018) 'Scutoids are a geometrical solution to three-dimensional packing of epithelia', *Nature communications*, 9(1), p. 2960. Available at: https://doi.org/10.1038/s41467-018-05376-1.

Gómez-Gálvez, P. *et al.* (2022) 'A quantitative biophysical principle to explain the 3D cellular connectivity in curved epithelia', *Cell systems*, 13(8), pp. 631–643.e8. Available at: https://doi.org/10.1016/j.cels.2022.06.003.

Guevorkian, K. and Maître, J.-L. (2017) 'Micropipette aspiration: A unique tool for exploring cell and tissue mechanics in vivo', *Methods in cell biology*, 139, pp. 187–201. Available at: https://doi.org/10.1016/bs.mcb.2016.11.012.

Ichbiah, S. *et al.* (2023) 'Embryo mechanics cartography: inference of 3D force atlases from fluorescence microscopy', *Nature methods*, 20(12), pp. 1989-1999. Available at: https://doi.org/10.1038/s41592-023-02084-7.

Navis, A. and Bagnat, M. (2015) 'Developing pressures: fluid forces driving morphogenesis', *Current opinion in genetics & development*, 32, pp. 24–30. Available at: https://doi.org/10.1016/j.gde.2015.01.010.

Sánchez-Gutiérrez, D. *et al.* (2016) 'Fundamental physical cellular constraints drive self-organization of tissues', *The EMBO journal*, 35(1), pp. 77–88. Available at: https://doi.org/10.15252/embj.201592374.

Sugimura, K., Lenne, P.-F. and Graner, F. (2016) 'Measuring forces and stresses in situ in living tissues', *Development* , 143(2), pp. 186-196. Available at: https://doi.org/10.1242/dev.119776.

Second decision letter

MS ID#: DEVELOP/2023/202362

MS TITLE: Local and global changes in cell density induce reorganisation of 3D packing in a proliferating epithelium.

AUTHORS: Vanessa Barone, Antonio Tagua, Jesus Á. Andrés-San Román, Amro Hamdoun, Juan Garrido-García, Deirdre C. Lyons, and Luis M. Escudero

I have now received all the referees reports on the above manuscript, and have reached a decision. The referees' comments are appended below, or you can access them online: please go to BenchPressand click on the 'Manuscripts with Decisions' queue in the Author Area.

© 2024. Published by The Company of Biologists under the terms of the Creative Commons Attribution License (https://creativecommons.org/licenses/by/4.0/).

The reviewers are all in favor of publication. Yet before I may proceed could you please address the minor issues mentioned by reviewer 2 and explain your response in the cover letter.

Reviewer 1

Advance summary and potential significance to field

I am happy with the revisions.

Comments for the author

The manuscript is clear and has interesting results. Further work is not necessary.

Reviewer 2

Advance summary and potential significance to field

In my earlier remarks, I mentioned that the transient occurrence of apico-basal T1 transitions (AB-T1s) in connection to cell divisions is an important new finding.
In addition, thanks to the changes mad by the authors, I now better understand what the authors mean by compaction, and I am convinced that it, too, can contribute to AB-T1s, which is an important contribution to the field.

Comments for the author

only minor comments:
- (Just a remark.) It seems to me that the appearance of AB-T1s "under compaction" is intimately linked to the pseudostratified nature of the epithelium, suggested by the new figure 4. Would the authors agree?

- The "surface ratio" is nowhere defined - I only realized that the apical to basal surface ratio is meant from the authors' reply. The definition is currently missing in the methods part. It is particularly easy to misinterpret it as there is also a "surface ratio anisotropy". Also, being more specific (e.g. apico-basal cell surface ratio) may help.

- Regarding the explanation of the Voronoi construction: This explanation is now improved. However, there is still a confusing aspect about it. On lines 626-628 and 631, the authors talk about the binarized mask, and suggest that it constrains the Voronoi construction. Yet, on lines 636-641, they seem to apply the mask again. This is confusing. What I believe is happening is that a Voronoi tesselation of the cell centroids is first applied without constraints. This leads to cells that do not have defined boundaries apically and basally. To define these boundaries, in a second step, the procedure in lines 636-641 is applied. Is this correct? If so, lines 626-628 and 631 are confusing, as they are not involved in the fist step of the construction. Similarly, the formulation "extraction of the outer and inner layers" is somewhat unclear; "definition of apical and basal sides of the cells" might be clearer.

- In the Voronoi constructions: are there Voronoi cells that do not extend to the apical or basal side of the epithelium? If this occurs, it should be more frequent for higher area number densities and cell heights. If such cells exist in the Voronoi constructions, do they also exist in vivo? Could such cells distort the AB-T1 statistics obtained?

Reviewer 3

Advance summary and potential significance to field

The concerns raised have been appropriately addressed by the authors, and they deserve congratulations this original contribution.

© 2024. Published by The Company of Biologists under the terms of the Creative Commons Attribution License (https://creativecommons.org/licenses/by/4.0/).

Comments for the author

The concerns raised have been appropriately addressed by the authors, and they deserve congratulations this original contribution.

Second revision

Author response to reviewers' comments

In this new version we have addressed the final comments from reviewer 2 as follows:

- (Just a remark.) It seems to me that the appearance of AB-T1s "under compaction" is intimately linked to the pseudostratified nature of the epithelium, suggested by the new figure 4. Would the authors agree?

We want to make clear that the epithelium is not pseudostratified. As can be seen in Fig. 1D, 3C or 3D, these epithelial cells can be considered “cuboidal”. The yellow cells in the bottom panel of figure 4 are a 2D lateral representation of scutoids.

- The "surface ratio" is nowhere defined - I only realized that the apical to basal surface ratio is meant from the authors' reply. The definition is currently missing in the methods part. It is particularly easy to misinterpret it as there is also a "surface ratio anisotropy". Also, being more specific (e.g. apico-basal cell surface ratio) may help.

We have now added the definition of surface ratio measurements in the method section.

- Regarding the explanation of the Voronoi construction: This explanation is now improved. However, there is still a confusing aspect about it. On lines 626-628 and 631, the authors talk about the binarized mask, and suggest that it constrains the Voronoi construction. Yet, on lines 636-641, they seem to apply the mask again. This is confusing. What I believe is happening is that a Voronoi tesselation of the cell centroids is first applied without constraints. This leads to cells that do not have defined boundaries apically and basally. To define these boundaries, in a second step, the procedure in lines 636-641 is applied. Is this correct? If so, lines 626-628 and 631 are confusing, as they are not involved in the fist step of the construction. Similarly, the formulation "extraction of the outer and inner layers" is somewhat unclear; "definition of apical and basal sides of the cells" might be clearer.

We have further clarified the procedure used to generate Voronoi constructions by modifying text in the relevant methods paragraph. The code used for the procedure is also publicly available.

- In the Voronoi constructions: are there Voronoi cells that do not extend to the apical or basal side of the epithelium? If this occurs, it should be more frequent for higher area number densities and cell heights. If such cells exist in the Voronoi constructions, do they also exist in vivo? Could such cells distort the AB-T1 statistics obtained?

All virtual cells in the Voronoi constructions extend to both the apical and basal sides of the tissue. This is what happen on the real epithelium, so the model mimics this feature.

Third decision letter

MS ID#: DEVELOP/2023/202362

MS TITLE: Local and global changes in cell density induce reorganisation of 3D packing in a proliferating epithelium.

© 2024. Published by The Company of Biologists under the terms of the Creative Commons Attribution License (https://creativecommons.org/licenses/by/4.0/).

AUTHORS: Vanessa Barone, Antonio Tagua, Jesus Á. Andrés-San Román, Amro Hamdoun, Juan Garrido-García, Deirdre C. Lyons, and Luis M. Escudero
ARTICLE TYPE: Research Article

I am happy to tell you that your manuscript has been accepted for publication in Development, pending our standard ethics checks.

© 2024. Published by The Company of Biologists under the terms of the Creative Commons Attribution License (https://creativecommons.org/licenses/by/4.0/).
